# Human‐Induced Pluripotent Stem Cells Generate Light Responsive Retinal Organoids with Variable and Nutrient‐Dependent Efficiency

**DOI:** 10.1002/stem.2883

**Published:** 2018-08-13

**Authors:** Dean Hallam, Gerrit Hilgen, Birthe Dorgau, Lili Zhu, Min Yu, Sanja Bojic, Philip Hewitt, Michael Schmitt, Marianne Uteng, Stefan Kustermann, David Steel, Mike Nicholds, Robert Thomas, Achim Treumann, Andrew Porter, Evelyne Sernagor, Lyle Armstrong, Majlinda Lako

**Affiliations:** ^1^ Newcastle University, Institute for Genetic Medicine Newcastle upon Tyne UK; ^2^ Newcastle University, Institute of Neuroscience Newcastle upon Tyne UK; ^3^ Merck KGaA Darmstadt Germany; ^4^ Novartis Basel Switzerland; ^5^ F. Hoffmann‐La Roche Ltd University of Tübingen Basel Switzerland; ^6^ Newcells Biotech Newcastle upon Tyne UK; ^7^ Centre for Biological Engineering Loughborough University Loughborough UK; ^8^ Newcastle University, Newcastle University Protein and Proteome Analysis Newcastle upon Tyne UK

**Keywords:** iPS, Retina, Retinal photoreceptors, Stem cells

## Abstract

The availability of in vitro models of the human retina in which to perform pharmacological and toxicological studies is an urgent and unmet need. An essential step for developing in vitro models of human retina is the ability to generate laminated, physiologically functional, and light‐responsive retinal organoids from renewable and patient specific sources. We investigated five different human‐induced pluripotent stem cell (iPSC) lines and showed a significant variability in their efficiency to generate retinal organoids. Despite this variability, by month 5 of differentiation, all iPSC‐derived retinal organoids were able to generate light responses, albeit immature, comparable to the earliest light responses recorded from the neonatal mouse retina, close to the period of eye opening. All iPSC‐derived retinal organoids exhibited at this time a well‐formed outer nuclear like layer containing photoreceptors with inner segments, connecting cilium, and outer like segments. The differentiation process was highly dependent on seeding cell density and nutrient availability determined by factorial experimental design. We adopted the differentiation protocol to a multiwell plate format, which enhanced generation of retinal organoids with retinal‐pigmented epithelium (RPE) and improved ganglion cell development and the response to physiological stimuli. We tested the response of iPSC‐derived retinal organoids to Moxifloxacin and showed that similarly to in vivo adult mouse retina, the primary affected cell types were photoreceptors. Together our data indicate that light responsive retinal organoids derived from carefully selected and differentiation efficient iPSC lines can be generated at the scale needed for pharmacology and drug screening purposes. stem cells *2018;36:1535–1551*

**Significance Statement**

Hallam and colleagues show herein that generation of retinal organoids from human iPSC is efficient, reliable and scalable. Using statistical models based on factorial experimental design, the authors have identified starting cell number and nutrient availability as key determinants for generation of laminated retinal organoids. The differentiation protocol was adapted to a multiwell plate format which enhanced generation of retinal organoids with retinal pigment epithelium (RPE) and improved ganglion cell development and the response to physiological stimuli. The authors show that the human iPSC derived retinal organoids are electrophysiologically functional and can be used as a viable alternative to animal models for toxicology testing.

## Introduction

Visual impairment affects 285 million people worldwide, with 90% of cases arising from chronic diseases 1. Approximately 80% arise in the aged population, and with a continued increase in life expectancy, the numbers affected by vision loss are set to rise 2, 3. As the retina does not readily regenerate, diseases such as age‐related macular degeneration (AMD), retinitis pigmentosa (RP), and glaucoma cause cell loss and result in irreversible profound visual impairment. For treatments to be developed, in vitro models of human retina that encompass all the retinal cell types and which are electrophysiologically responsive to light are required to screen novel substances for pharmacological and toxicological effects. Most drug studies are currently performed in vivo using rodent models, but this approach is far from optimal because there are fundamental structural and functional differences between rodent and human retina. To date, there are no published adequate in vitro models that incorporate the diversity of all retinal cell types and that can be maintained stably in culture for prolonged periods of time. Simple models such as organotypic retinal explants have been prepared from embryonic and postnatal rodents. However, retinal ganglion cells (RGCs) degenerate 4, photoreceptors do not grow extended outer segments 5, amacrine cells are lost gradually over time 6, and the expression of proteins in the interphotoreceptor matrix is significantly reduced when compared with normal adult retina 7. Most animal models are also limited by anatomical differences such as the absence of a fovea in all mammals except for simian primates. Other widely used in vitro cell line models lack efficacy, are variable in morphology and limited in the recapitulation of the composition of the retina 8, 9, 10.

Recent studies have shown that organoids generated from tumors or pluripotent stem cells can compositionally resemble primary tissue, producing multiple cell lineages while maintaining the convenience of unlimited expanding capacity. The use of organoids has been successfully translated in diseases such as Alagille syndrome, whereby organoids mimic the in vivo disease pathology 11. In addition, they have been used in cancer screening and mapping mutational signatures in CRISPR modified organoids 12. The use of human pluripotent stem cells for generating isolated retinal cell types as well as completely laminated retina has been published 13, 14, 15, 16, 17, 18, 19, 20, 21, 22, 23, 24, 25, 26, 27, 28, 29, 30, 31, 32. Three‐dimensional (3D) culture in particular seems to enhance the maturation of multi‐layered or laminated structures, which recapitulate human retinogenesis and generate transplantable photoreceptors 13, 14, 17, 21, 31, 32. To date, retinal organoids have not been extensively used in toxicology or pharmacology screening programs, largely due to lack of differentiation methods that generate light responsive 3D retinae closely matching the mature retina in sufficiently large numbers needed for this type of assay.

In this article, we investigated the ability of five different human iPSC lines to generate laminated retinae and their responses to light and other chemical stimuli. We applied statistical design of experiment (DoE) methodology to determine multiple variables critical for process control and successfully adapted the differentiation protocol to a 96‐well format, which enables large‐scale automation and drug screening. We performed proof of concept studies and investigated the response of retinal organoids to a known retinotoxic agent (Moxifloxacin) to validate their usefulness for toxicity screening programs.

## Materials and Methods

### Cell Culture

iPSC lines, WT1 (Female, aged 31) 33, WT2 (Male, aged 68) 34, WT3 (Female, aged 84) 34, RP (PRPF 31, c.1115_1125del11, RP11VS iPSC line) 33, and AMD (CFH, rs1061170, F181 iPSC line) 35 (derived and fully characterized) were cultured with mTeSR1 (Stem Cell Technologies, Cambridge, UK) on growth factor reduced matrigel‐coated 6‐well plates (Corning, NY). Retinal organoids were generated by dissociating cells at 90% confluence with Accutase (Thermo Fisher Scientific, Waltham, MA) and seeding 3,000–12,000 cells into each well of a 96‐well lipidure‐coated U‐bottom plate (Amsbio, Boston, MA) in 100 μl of mTeSR1 with 10 μM ROCK inhibitor (Y27632, Tocris, Bristol, UK). After 2 days, mTeSR1 + ROCK inhibitor was changed to differentiation medium, which comprised 45% IMDM (225 ml), 45% HAM's F12 (225 ml), 10% KOSR (50 ml), 1% GlutaMAX (5 ml), 1% Chemically Defined Lipid Concentrate (11905031; 5 ml), Pen/Strep (5 ml; Thermo Scientific, UK) and 1‐thioglycerol M6145 (450 μM; Sigma, UK). Medium was changed every 2 days, at day 6 BMP‐4 (1.5 nM) was added at day 6 as described in Kuwahara et al. 36 At day 18, “pooled” retinal organoids were transferred to ultra‐low attachment 6‐well plates (Corning, NY) and medium was changed to maintenance media: DMEM/F12, 10% FBS, GlutaMAX (5 ml), 1% N2, Pen/Strep (5 mL), plus 3 μM CHIR99021 (Stem Cell Technologies, Cambridge, UK), 5 μM SU5402 (Bio‐techne, Abingdon, UK). At day 24, medium was changed to maintenance media minus CHIR99021 and SU5402.

Process control experiments where performed as described above, with the following modifications in design 1: cell‐seeding number of 6,000, 9,000, or 12,000. KOSR: 5%, 10%, or 15%; chemically defined lipid concentrate: 0.5%, 1% or 1.5%; 1‐thioglycerol: 300, 450, or 600 μM; BMP4: 0.75, 1.5, or 2.25 nM. Design 2: cell number: 3,000, 5,500, or 8,000; CHIR99021: 1.5, 3, and 6 μM: SU5402: 2.5, 5, and 10 μM. BMP4: 2, 3.5, and 5 nM.

Moxifloxacin (Sigma, Cambridge, UK) was added to the culture media at concentrations of 50, 100, 300, 700, and 1000 μg/ml with an exposure time of 24 hours.

### RNA Extraction and qPCR

About 15–20 retinal organoids were homogenized using a Dounce Tissue Grinder (Sigma) and RNA was extracted using the Promega tissue extraction kit (Promega, Madison, WI) as per the manufactures instructions. About 1 μg of RNA was reverse transcribed using random primers (Promega). qRT‐PCR was performed using a Quant Studio 7 Flex system (Applied Biosystems, Foster City, CA) with SYBR Green (Promega). Each primer (listed in Supporting Information Table S1) was used at a concentration of 1 μM, and at a ratio of 50:50 for forward and reverse. The reaction parameters were as follows: 95°C for 15 minutes to denature the cDNA and primers, 40 cycles of 94°C for 15 seconds followed by primer specific annealing temperature for 30 seconds, succeeded by a melt curve. All primer pairs were validated on adult human retina. A comparative Ct method was used to calculate the levels of relative expression, whereby the Ct was normalized to the endogenous control (*GAPDH*). This calculation gives the ΔCt value, which was then normalized to WT1, giving the ΔΔCt. The fold change was calculated using the following formula: 2^−ΔΔCt^. Statistical significance analyses were evaluated using Prism (GraphPad, La Jolla, CA).

### Immunohistochemistry

Retinal organoids were collected at days 35, 90, and 150, washed with PBS for 10 minutes and then fixed in 4% paraformaldehyde for 15 minutes. Retinal organoids were washed again with PBS before being placed in 30% sucrose overnight. Retinal organoids were placed into cryogenic molds and immersed in optimum cutting temperature (OCT). About 10 μM slices were sectioned on a Leica cryostat (CM1850). Cut sections were blocked in 0.3% Triton‐X‐100 (Sigma) and 10% NGS (Thermo Scientific) in PBS for 1 hour. This solution was removed and replaced with antibody diluent (0.3% Triton‐X‐100, 1% BSA in PBS) with applicable antibody dilution (Supporting Information Table S2) at 4°C overnight. Sections were washed three times in PBS followed by incubation with secondary antibodies for 2 hours at room temperature. Sections were washed again as stated previously and mounted in Vecta‐shield (Vector Labs, Burlingame, CA) with Hoechst 33342 (1:1000, Thermo Scientific) or DAPI (Sysmex, 1:1000). Images were captured using Nikon A1R confocal (resonant, invert; Nikon, Japan) and Zeiss Axio ImagerZ2 equipped with an Apotome2 (Zeiss, Germany).

### Quantitative Assessment of Retinal Determination in 3D Culture

Laminated optic vessels and pigmented patches were recorded in both 96‐well formats and in 6‐well plates using high‐resolution (3,200 dots per inch) scanned images (Epson Perfection V500, Japan) at day 35, 90, and 150 of differentiation, confirmed with representative brightfield imaging (Supporting Information Fig. S1; AxioVert, Zeiss Germany). In addition, immunostaining of representative examples was carried out with (AxioImager with Apotome, Zeiss Germany) with RPE, photoreceptor and ganglion/amacrine cell markers to determine presence of at least two recognizable layers in the retinal organoids. Criteria for counting were as follows: the presence of bright neuroepithelium at the apical edge of retinal organoids showing immunostaining for two different retinal layers were counted as neural retina (NR), pigmented patches showing immunostaining for RPE markers were counted as RPE and organoids with both neural retina and RPE as NR + RPE. Organoids in which the retinal structure was not clear to the operator and could not be confirmed with immunostaining were counted as undefined. Counts of organoids with and without RPE and/or neural retina were performed by two separate investigators and did not deviate significantly from one another. In total, 288 organoids per condition (single or pooled) were analyzed bringing the total number of organoids scored per iPSC line to 576, representing three biological replicates. ANOVA analysis showed a significant difference between the iPSC lines to generate retinal organoids with or without RPE at days 35, 60, and 90 of differentiation (*p* < .05). The same analysis performed within the same cell line (biological replicates) showed the variability to be insignificant at all differentiation timepoints examined (*p* > .05).

### LDH Cytotoxicity Test

Lactate dehydrogenase (LDH; Pierce LDH Cytotoxicity Assay Kit, Thermo Scientific) released by dead/dying cells was detected by incubating cell culture supernatant with lactate, which is converted to pyruvate in the presence of LDH and NAD^+^. NAD^+^ is converted to NADH Diaphorase and uses NADH to reduce tetrazolium salt (INT) to a red formazan product that can be measured at 490 nm using a Varioskan Lux (Thermo) plate reader. Validated positive control was supplied in kit and suspended in 1% BSA.

### Electrophysiological Recordings

Experimental procedures on neonatal mice were approved by the ethical committee at Newcastle University and carried out in accordance with the guidelines of the UK Home Office, under control of the Animals (Scientific Procedures) Act 1986. Organoids and neonatal retinas were transferred to 33°C artificial cerebrospinal fluid (aCSF) containing the following (in mM): 118 NaCl, 25 NaHCO_3_, 1 NaH_2_PO_4_, 3 KCl, 1 MgCl_2_, 2 CaCl_2_, 10 glucose, and 0.5 l‐Glutamine, equilibrated with 95% O_2_ and 5% CO_2_. Organoids were opened longitudinally and placed, with the presumed RGC layer facing down, onto the 4096 channel MEA, flattened with a translucent polyester membrane filter (Sterlitech Corporation, Kent, WA). The organoids were allowed to settle for at least 2 hours. Mice aged between postnatal days (P) 10–12 were dark‐adapted overnight and sacrificed by cervical dislocation. Retinas were isolated from the eye cups and flattened for MEA recordings 39. Recordings were performed on the BioCam4096 multielectrode array (MEA) platform with BioChips 4096S+ (3Brain GmbH, Lanquart, Switzerland), integrating 4096 square microelectrodes in a 64 × 64 array configuration. To reliably extract spikes from the raw traces, we used a quantile‐based event detection 37 and single‐unit spikes were sorted using an automated spike sorting method for dense, large‐scale recordings 38. Statistical significance and firing rate analyses were evaluated using Prism (GraphPad) and MATLAB (MathWorks, Natick, MA). Light stimuli were projected as described previously 39. To yield maximal responses to light stimuli in organoids, broad white (high photopic) light pulses (pulsed light: 200 ms, 217 μW/cm^2^ irradiance, 1 Hz) were flashed for 5 min onto the organoids following recording spontaneous activity in the dark for 5 min. We also used full field stimuli (in the mesopic light range) that switched from light to dark (0.5 Hz, 30 repetitions, 4 μW/cm^2^ irradiance) to calculate peristimulus time histograms (bin size = 25 ms), allowing us to isolate light responses from spontaneous activity in neonatal retinas. To sort RGC responses according to their main response polarity, we measured the relative amplitude of ON (during light; A1) and OFF (during dark; A2) responses and calculated the bias index (BI) defined as (A1 − A2)/(A1 + A2) 58. We used the BI to split the organoid and neonatal mouse retinal responses into OFF (BI −1 to −0.15) and ON cells (BI 0.15–1). The drugs cGMP (8‐Bromoguanosine 3′,5′‐cyclic monophosphate, Sigma‐Aldrich, St. Louis, MO) and GABA (γ‐aminobutyric acid, Tocris Bioscience, Bristol, UK) were puffed in the recording chamber (final concentrations, cGMP 100 μM, GABA 125 μM) and activity was recorded continuously for 4 minutes, starting at 2 minutes before the puff.

### Process Control

DoE methodology (Minitab v18 Software, Minitab Inc., State College, PA) was applied to evaluate the effects of key culture medium components on lineage indicative gene expression. An initial full factorial design, with duplicate center points to estimate error, was applied to screen the effects of initial cell density (6,000 and 12,000), 1‐thioglycerol (300 and 600 μM), BMP4 (0.75 and 2.25 nM), KSR (5% and 15%), and Lipids (0.5% and 1.5%). A subsequent duplicated ½ fraction factorial design with center points was applied to evaluate influential factors BMP4 (2 nM, 5 nM) and cell density (3,000 and 8,000) at improved ranges identified from the initial design and in conjunction with CHIR99021 (1.5 and 6 μM) and SU5402 (2.5 and 10 μM). DoE responses were CT for the target gene normalized to GAPDH (CT GAPDH – CT Target Gene). In each case, multiple regression was applied to fit a general linear model for each response followed by model reduction through hierarchically removing predictor terms that had a statistical significance of *p* ≥ .05 (ANOVA). Residual plots were evaluated for normal distribution and no systematic trends with respect to experimental order or fitted value.

### Transmission Electron Microscopy

Cells were fixed with 2% glutaraldehyde and kept at 4°C. TEM including all the cell processing was performed at Newcastle University Electron Microscopy Research Services, Ultrathin sections were stained with heavy metal salts (uranyl acetate and lead citrate) and imaged on a Philips CM100 TEM (Philips, Minato‐ku, Japan).

### Secretome Analysis

To process secretome samples, supernatant samples were run on a 4–12% SDS PAGE and treated with silver nitrate (AgNO_3_), lanes representing untreated control supernatant, moxifloxacin (100 μM) treated supernatant were excised, then separated into 10 sections. Gel pieces were destained (15 mM K_3_Fe(CN)_6_ and 50 mM Na_2_S_2_O_3_) then proteins reduced with 10 mM dithiothreitol and alkylated with 50 mM iodoacetamide. Proteins were digested by the addition of trypsin added at a ratio of 30:1 (protein:trypsin) and incubated for 16 hours at 37°C. Peptides were extracted from the gel pieces with increasing concentrations of acetonitrile and the resulting peptide solutions were concentrated by vacuum evaporation and desalted using home packed C18 stage tips 40. Each peptide sample was analyzed in a separate LCMS experiment using a Thermo RSLC Nano HPLC system coupled to a Sciex 6600 mass spectrometer. Peptides were loaded onto a 300 μm Prepmap trap column (Thermo) before being resolved on a 23 cm 75 μm ID home‐packed analytical column containing Dr Maisch 3 μm particle size stationary phase. Peptides were injected online into the mass spectrometer using data‐dependent acquisition. Survey scans were performed over an *m*/*z* range of 400–1200. From each survey, the 30 most intense ions were selected for MS/MS. Precursors were fragmented with a rolling collision energy, based on the charge state of the peptide ion. Total cycle time was 1.7 seconds.

Raw data was converted to .mgf peaklists using MSconvert 41. These were then searched against the human proteome (Uniprot, entry version 139 updated 07/07/2017) and cRAP (version 01/01/2012) 42 with the reversed fasta files for both using X!Tandem 43. The following parameters: search tolerance MS, 20 ppm. MS/MS fragment ions 30 ppm, fixed modification of +57.0215@C. The following modifications were considered in a second pass search (refinement), 0.98401@N, 79.9663@S, 79.9663@T, 79.9663@Y, 15.994915@M, 15.994915@W, 0.98401@Q, 14.016@C, 14.016@D, 14.016@E, 14.016@H, 14.016@R, 14.016@K, 15.994915@K, and 15.994915@P. Alternatively, raw files were processed and searched using MaxQuant 44 using the following parameters; human proteome (Uniprot, entry version 139 updated 07 July 2017), Label Free Quantitation‐LFQ, iBAQ, Fixed modifications: carbamidomethyl (C), variable modifications oxidation (M), and acetyl (protein N‐term), TOF MS/MS match tolerance 40 ppm. Output files were loaded into Perseus 45 for statistical evaluation and hierarchical clustering comparison, then a visual heatmap representation of the clustered matrix was generated.

## Results

### iPSC Lines Generate Retinal Organoids with Variable Efficiency

**Figure 1:**
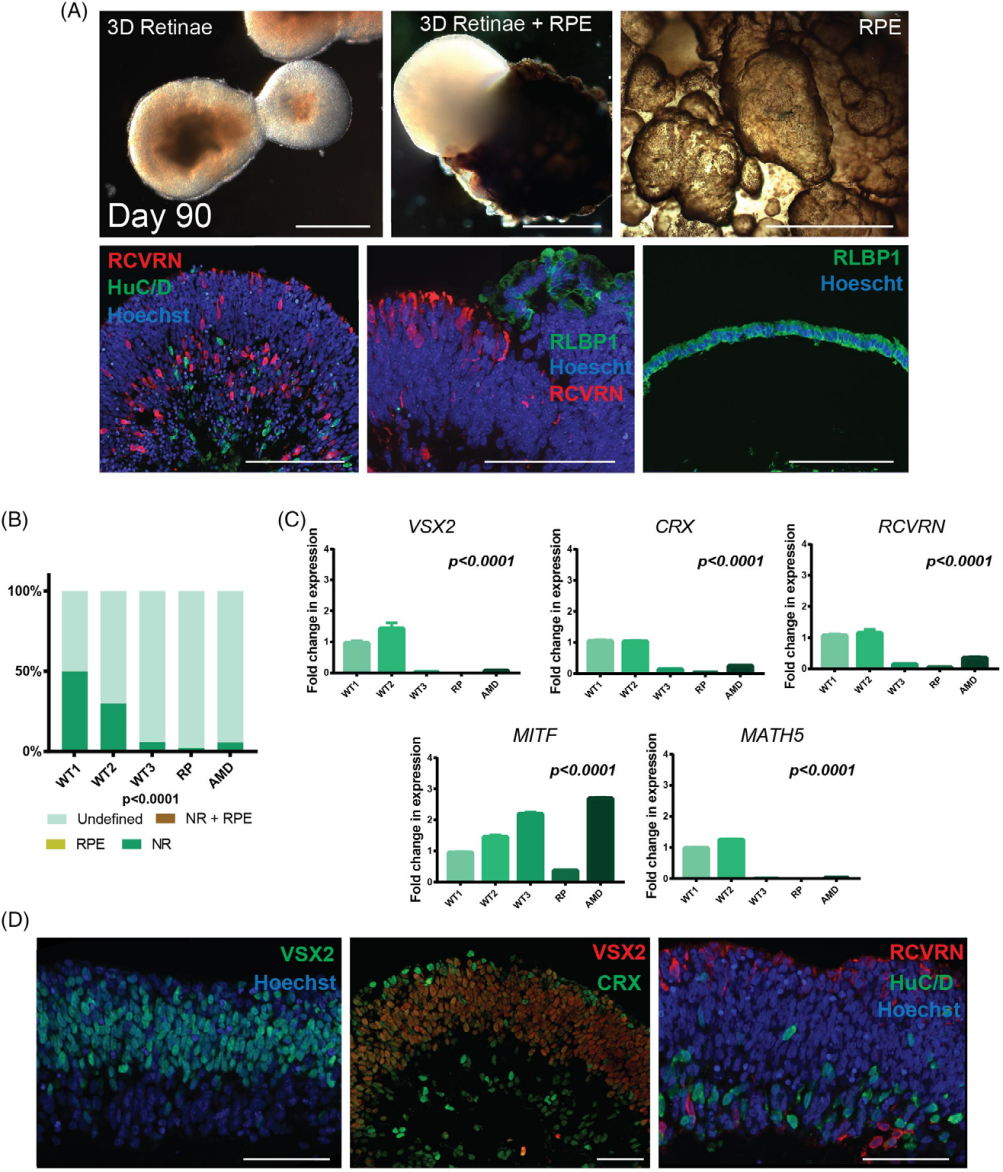
Characterization of iPSC derived retinal organoids at day 35 and 90 of differentiation. **(A):** Representative examples of iPSC derived retinal organoids (with and without RPE) and RPE spheres. Left top, light microscopy images of retinal organoids without RPE (scale bar = 0.5 mm). Middle top, retinal organoid with RPE (scale bar =0.5 mm). Right top, RPE spheres showing pigmentation and hollow center (scale bar = 100 μm). (**A**) Left bottom, photoreceptor marker RECOVERIN & ganglion and amacrine cell marker HuC/D immunofluorescence (scale bar = 100 μm). Middle bottom and right, RPE and Muller glia cell marker RLBP1 (scale bar = 100 μm). **(B):** Schematic chart showing the development of iPSC derived retinal organoids and RPE spheres, *n* = 3. Abbreviations: RPE, RPE spheres; NR, retinal organoids with neural retina (no RPE); NR+ RPE, retinal organoids with RPE; undefined, organoids that did not contain any neural retina or RPE cells. **(C)**: RT‐PCR expression analysis of iPSC derived organoids. *n = 3,* error bars = SEM. Significance assessed by one way ANOVA with Tukey's multiple comparisons test. All results are shown relatively to WT1; **(D):** Example immunofluorescence staining of VSX2, CRX, RCVRN, HuC/D, and Hoechst in day 35 retinal organoids (scale bar = 50 μm).

To investigate the ability of fibroblast‐derived iPSC lines to generate retinal organoids, we differentiated three fully characterized iPSC lines from unaffected donors (referred thereafter as WT1, WT2, and WT3) 34, one iPSC line from a donor with age related macular degeneration (referred thereafter as AMD) 35 and one iPSC line from a subject with retinitis pigmentosa (referred thereafter as RP 33 using the induction‐reversal protocol developed by Kuwahara et al. in hESC for cogeneration of neural retina and RPE 36. We applied two small changes to this protocol, including addition of Y26732 (10 μM; Rho kinase inhibitor) for the first 48 hours of differentiation to enhance aggregate formation and U‐shape bottom plates which are better suited for imaging and used by other groups for retinal differentiation of iPSCs 32. The relative organoid size (Supporting Information Fig. S2) and the emergence of laminated neural retina and retinal pigmented epithelial (RPE) was assessed at several time points during the differentiation process (Fig. 1A). Formation of RPE spheres which were hollow inside and composed of a single layer of RLBP1 positive hexagonal pigmented RPE cells (Fig. 1A) was observed during the differentiation of some of the iPSC lines. At day 35, the efficiency of generating retinal organoids and the organoid size was variable between the iPSC lines (Fig. 1B and Supporting Information Fig. S2). Furthermore, no RPE was detected at this stage in organoids from any of the five iPSC lines (Fig. 1B). This variability was corroborated by the gene expression analysis which indicated that the iPSC lines which were more efficient at giving rise to retinal organoids (WT1 and WT2) had higher expression of photoreceptor progenitor (*VSX2*), photoreceptor precursor (*CRX* and *RCVRN*), and retinal ganglion cell (*MATH5*) markers compared with the less efficient iPSC lines (Fig. 1C). Although no apparent RPE was observed in the organoids from any of the five iPSC lines at this time point, the expression of RPE marker (*MITF*) was higher in the two iPSC lines which were more efficient at giving rise to RPE (WT3 and AMD) during the course of differentiation. Immunocytochemical analysis of retinal organoids at this time point showed that the large majority of cells situated in the apical layer of the retinal organoids were positive for the retinal progenitor marker VSX2. A smaller percentage of cells were also positive for CRX, which indicates the emergence of photoreceptor precursors alongside presumptive RGC and/or amacrine cells (marked by HuC/D expression) in the basal layer (Fig. 1D). Recoverin, a marker of photoreceptor precursors and a subset of bipolar and ganglion cells was observed at the apical layer and less so in the basal layer of the organoids.

**Figure 2:**
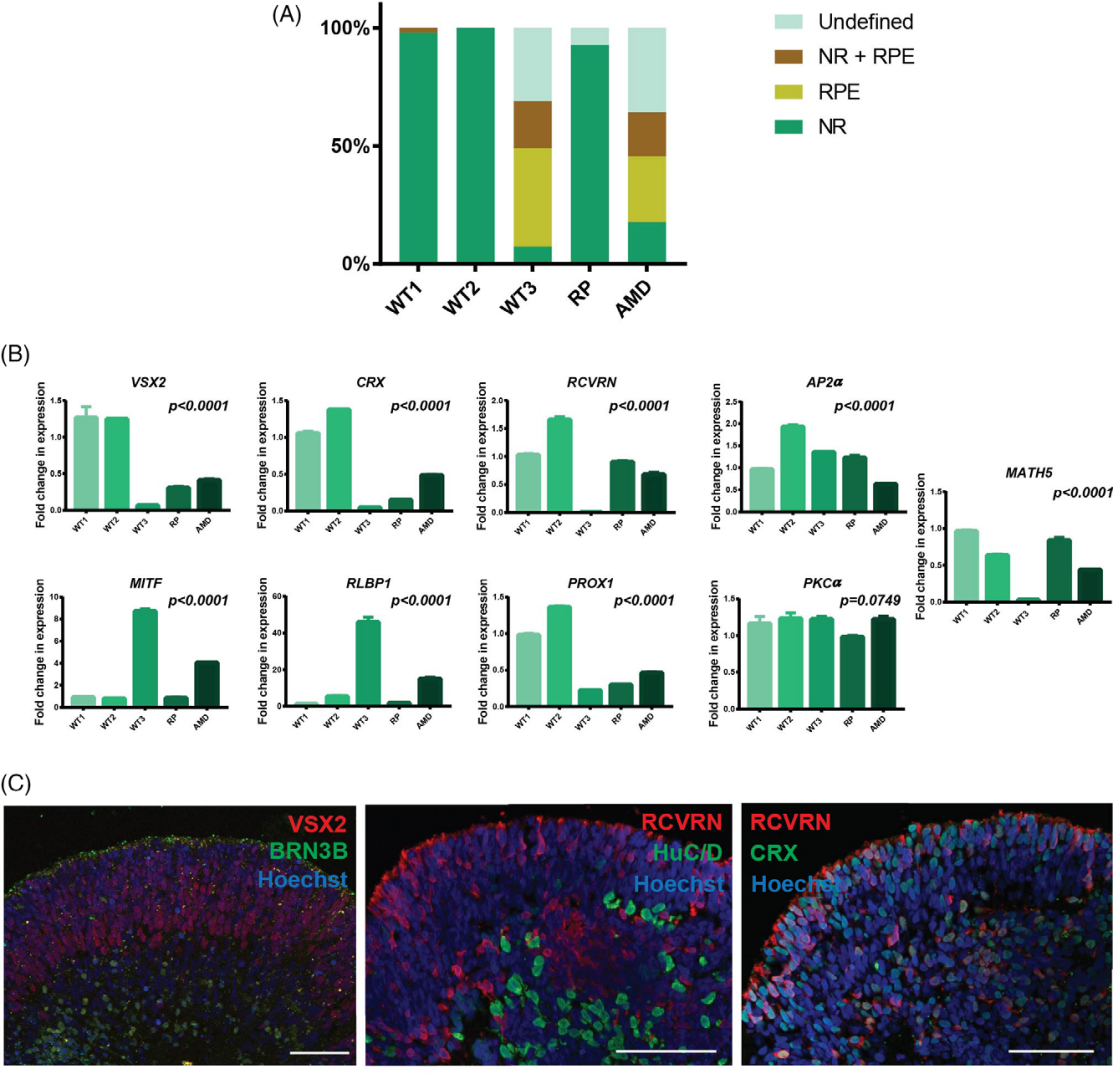
Characterization of iPSC derived retinal organoids at day 90 of differentiation. **(A):** Schematic chart showing the development of iPSC derived retinal organoids and RPE spheres, *n = 3.* Abbreviations: RPE, RPE spheres; NR, retinal organoids with neural retina (no RPE), NR + RPE, retinal organoids with RPE; undefined, organoids that did not contain any neural retina or RPE cells. **(B):** RT‐PCR expression analysis of iPSC derived organoids. *n = 3*, error bars = SEM. Significance assessed by one way ANOVA with Tukey's multiple comparisons test. All results are shown relatively to WT1; **(C):** Example immunofluorescence staining of VSX2, BRN3B, RCVRN, HuC/D CRX, and Hoechst in day 90 retinal organoids (scale bar = 50 μm).

By day 60, RPE was observed more prominently in two of the iPSC lines (WT3 and AMD), both of which showed the highest propensity to form RPE spheres and retinal organoids with RPE (Supporting Information Fig. S3). The relative size of organoids was also variable at this time point (Supporting Information Fig. S2), most likely reflecting the heterogeneous nature of organoids across the iPSC lines. By day 90, we observed a marked increase in the ability of iPSC to give rise to organoids containing neural retina with and without RPE (Fig. 2A), albeit the relative organoid size remained variable (Supporting Information Fig. S2). At this time point, there were significant differences in gene expression between iPSC lines for all markers studied (Fig. 2B). The iPSC lines which showed the highest commitment to form retinal organoids (WT1 and WT2; Fig. 2A) still showed higher expression of photoreceptor progenitor and precursor markers (*VSX2, CRX*, and *RCVRN*) and developing horizontal, bipolar, and amacrine cell marker (*PROX1*; Fig. 2B). The expression of RPE marker (*MITF*) and RPE and Müller Glia cell marker (*RLBP1*) was the highest in the organoids generated from the two iPSC lines with the highest commitment to RPE (WT3 and AMD; Fig. 2B). These two lines also showed the lowest expression of retinal ganglion cell marker (*MATH5*), which correlates with their lower efficiency in forming retinal organoids (Fig. 2B). Immunocytochemistry analysis showed prominent VSX2 in the apical layer and BRN3B in the basal layers of the retinal organoids, indicating formation the presence of photoreceptor progenitors and RGCs, respectively (Fig. 2C). Cells with elongated morphology reminiscent of developing photoreceptors, showing RCVRN and CRX coexpression, were clearly observed at this time point in the apical layer of the retinal organoids (Fig. 2C). HuC/D positive cells were observed in the basal layer of the organoids as well as adjacent to the outer nuclear like layer, suggesting the development of amacrine and ganglion cells.

**Figure 3:**
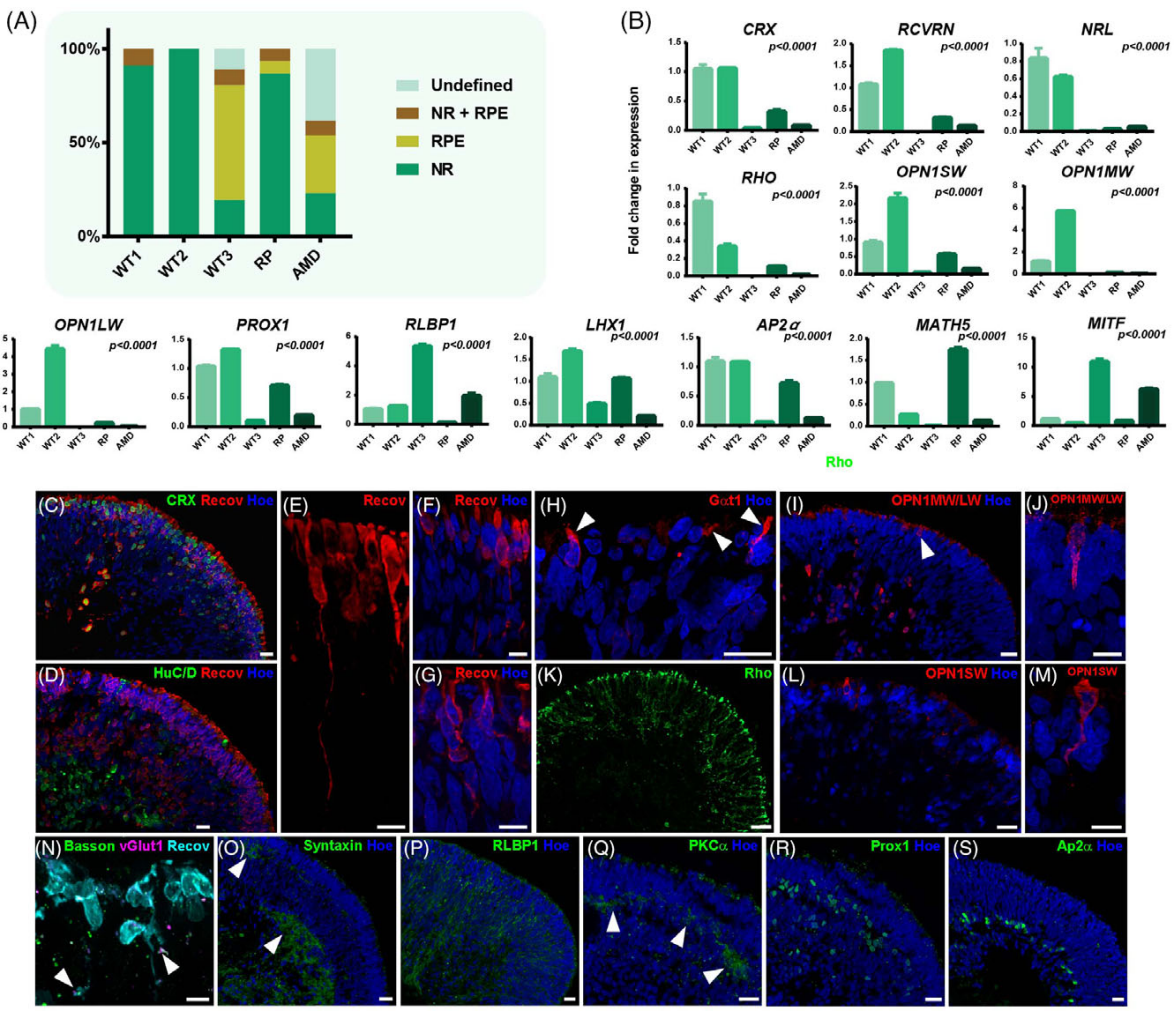
Characterization of iPSC derived retinal organoids at day 150 of differentiation. **(A):** Schematic chart showing the development of iPSC derived retinal organoids and RPE spheres, *n = 3.* Abbreviations: RPE, RPE spheres, NR, retinal organoids with neural retina (no RPE), NR+ RPE, retinal organoids with RPE; undefined = organoids that did not contain any neural retina or RPE cells. **(B):** RT‐PCR expression analysis of iPSC derived organoids. *n = 3*, error bars = SEM. Significance assessed by one way ANOVA with Tukey's multiple comparisons test. All results are shown relatively to WT1; **(C):** Example immunofluorescence staining of VSX2, BRN3B, CRX, RCVRN, HuC/D, RLBP1, AP2‐α, PKC‐α, PROX1, OPN1SW, BASSON, VGLUT1, and Hoechst in day 150 retinal organoids (scale bar = 50 μm). **(C):** Recoverin‐positive photoreceptors (Recov, red) are colocalized with Crx (green). **(D):** HuC/D‐positive amacrine and ganglion cells (green) form a separate layer in the center of organoids while photoreceptors (Recov, red) are on the apical site. **(E–G):** Higher magnifications of recover in expressing photoreceptors and their morphology (Recov, red) including putative outer segments and axon terminals. **(H):** Photoreceptor outer segments expressing Gαt1 (red; arrows). **(I):** OPN1MW/LW (red) is expressed in cones located on the apical site (arrow) as well as in the center of organoids. **(J):** Higher magnification of a middle/long wavelength cone (red) on the apical site of organoids. **(K):** Rhodopsin (Rho, green) expression was found on the apical site of organoids. **(L):** OPN1SW (red) is expressed in cone at the apical site of organoids. **(M):** Higher magnification of a short wavelength cone (red) on the apical site of organoids. **(N):** Bassoon (green), vGlut1 (magenta), and Recoverin (Recov, cyan) show colocalization at putative axon terminals (arrows). **(O):** Syntaxin (green) is expressed in two layers, one underneath the photoreceptor layer and the other more towards the center of organoids (arrows). **(P):** Müller cells (CRALBP, green) are spanning through the whole organoid. **(Q):** PKCα (green) is expressed underneath the photoreceptor layer in retinal organoids (arrows). **(R,S):** Horizontal cells, marked by Prox1 (R; green) are located slightly above Ap2α‐positive amacrine cells (S; green) in the middle of retinal organoids.

By day 150, 100% of the organoids for three of the iPSC lines (WT1, WT2, and RP) developed neural retina (with and without RPE) and RPE spheres (Fig. 3A); however, a fraction of organoids for the AMD and WT3 iPSC line at this stage did not develop neural retina like structures or RPE spheres and were classified as undefined. At this time point, there were no significant differences in organoid size (Supporting Information Fig. S2). It is of interest also to note that in all three lines derived from unaffected controls (WT1, WT2, and WT3), the increase in organoid size occurred till day 90 with negligible increase occurring between day 90 and day 150 (data not shown); however, this was not the case for the disease iPSC lines (RP and AMD) which showed a continued increase in size throughout the differentiation process (AMD iPSC line) or only between day 90 and 150 of differentiation (RP iPSC line). At this time point, the expression of photoreceptor precursor (*CRX and RCVRN*), rod precursor (*NRL*), cone (*OPN1SW, OPN1MW,* and *OPN1LW*), rod (*RHO*) as well as horizontal, amacrine, and bipolar cell markers (*PROX1, AP2‐α,* and *LHX1*) were still higher in the WT1 and WT2 lines (Fig. 3B). The expression of RPE marker (*MITF*) as well as Müller glia and RPE marker (*RLBP1*) remained highest in WT3 and AMD lines (Fig. 3B) which were more efficient at giving rise to RPE spheres. The retinal organoids derived from these two iPSC lines also showed the lowest expression of *MATH5*, a finding that most likely reflects the fact that a lesser percentage of organoids generated from these lines were able to form retinal organoids at this time point.

Immunocytochemistry analysis at day 150 showed that recoverin (a marker of photoreceptor and bipolar cells) expression was detected in the apical surface of the retinal organoids (Fig. 3C,D). Furthermore, cells with coexpression of RCVRN and CRX were found in the outer layer of the organoids (Fig. 3C). Nonetheless, a few cells coexpressing both markers were also located in the presumptive inner nuclear layer which is suggestive of interkinetic nuclear migration of photoreceptor progenitors noted during retinal development 46. HuC/D, a marker of ganglion and amacrine cells was found in the basal layer of the retinal organoids (Fig. 3D). High magnification of Recoverin positive cells highlighted the photoreceptor morphology with the presence of outer like segments and axons stratifying towards the putative outer plexiform layer (Fig. 3E–G). The photoreceptors residing in the apical layer were immunopositive for ARL13B expression (data not shown), indicating formation of connecting cilia and beginning of outer segments, which were marked in rod photoreceptors by the expression of Gα transferase 1 (Fig. 3H, arrows). The photoreceptor marker, OPN1MW/LW, was expressed in cones located on the apical site as well as in progenitors toward the center of organoids (Fig. 3I,J). Rhodopsin, a rod marker, was also located apically within the organoids (Fig. 3K). Meanwhile, another cone marker, OPN1SW (short wave) was expressed exclusively at the apical surface (Fig. 3L,M). Presynaptic active zone protein bassoon and vGlut1 a protein associated with synaptic vesicles showed colocalization at putative photoreceptor axon terminals (Fig. 3N, arrows). Syntaxin, another protein located at presynaptic active zones at nerve endings was observed in two locations, beneath the photoreceptor layer and basally indicating the formation of putative outer and inner plexiform like layers (Fig. 3O, arrows). CRALBP (also known as RLBP1), a marker of Müller glia cells spanned the entire organoid structure (Fig. 3P). PKC‐α positive cells were also detected beneath the photoreceptor layer in the organoids suggesting the emergence of rod bipolar cells (Fig. 3Q, arrows). PROX1, Prospero homeobox protein 1, a transcription factor involved in development, and shown to be expressed in the developing horizontal, bipolar, and amacrine cells, was detected in the middle layer of the organoids in two locations: adjacent to the presumptive outer nuclear layer as well as adjacent to the developing ganglion cell layer (Fig. 3R). AP2‐α is a marker of amacrine cells and belongs to a family of AP‐2 transcription factors, which respond to retinoic acid and are considered intrinsic to retinal development 47. AP2‐α positive cells were observed in the middle layer of day 150 organoids (Fig. 3S). Collectively, these findings suggest that the formation of an inner nuclear like layer in these retinal organoids.

In summary, our data suggest variabilities between iPSC lines with reference to overall efficiency in generating retinal organoids, kinetics of differentiation and the development of RPE within the organoids themselves. This variability cannot be related solely to disease pathology as it was encountered also between iPSC lines from unaffected individuals. Despite such variability, by month 5 of differentiation, all iPSC lines were able to generate laminated retinal organoids with well‐formed outer nuclear like layers containing photoreceptors with inner segments, connecting cilium and outer like segments.

### Adaptation of Retinal Differentiation Protocol to 96‐Well Plate Format

**Figure 4:**
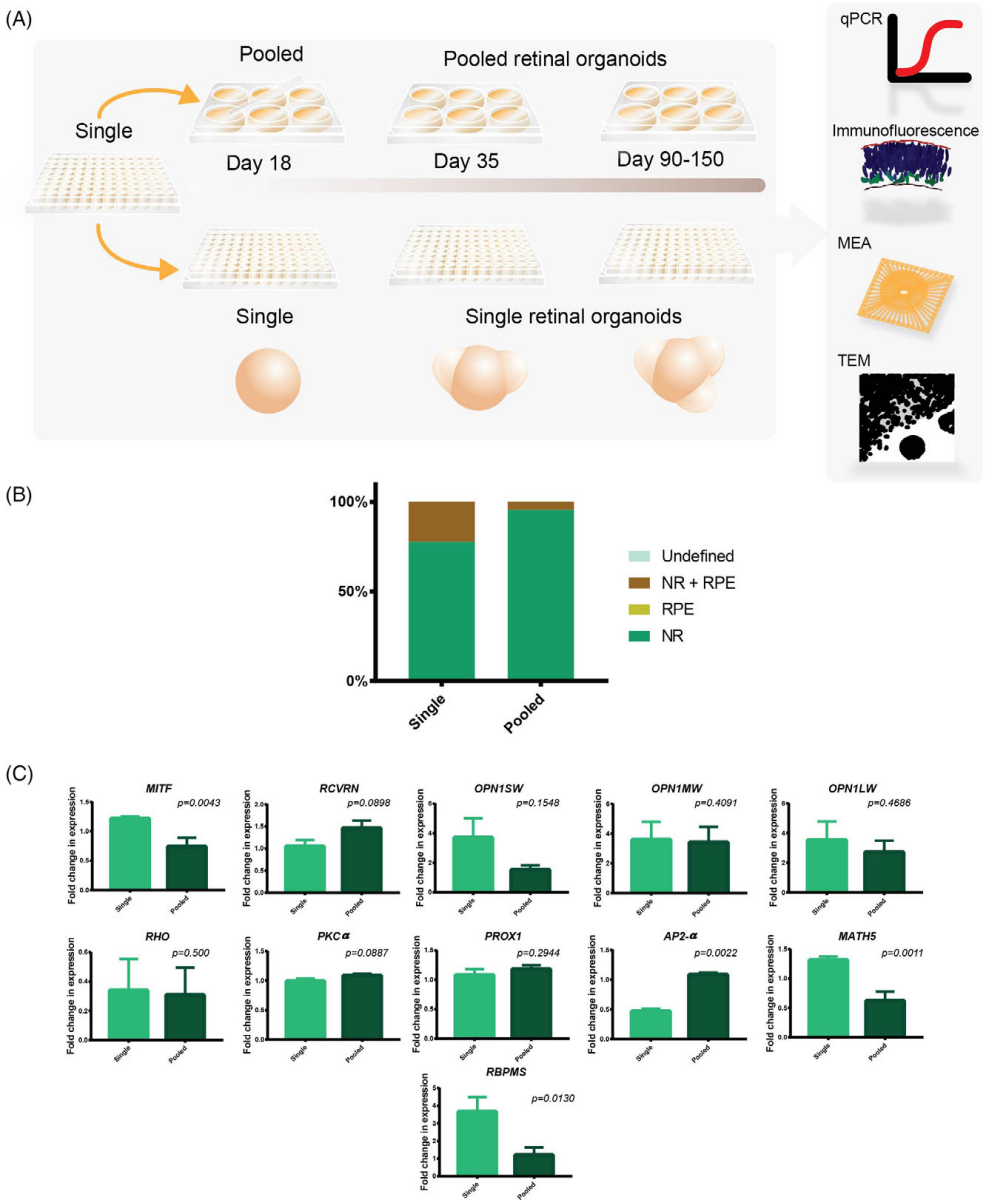
iPSC differentiation protocol can successfully be transferred to 96‐well plates. **(A):** Diagrammatic representation of the culture formats for both single and pooled retinal organoids. **(B):** Percentage of retinal structures compared between single and pooled conditions *n = 3.* Abbreviations: RPE, RPE spheres; NR, retinal organoids with neural retina (no RPE); NR+ RPE, retinal organoids with RPE; undefined, organoids that did not contain any neural retina or RPE cells. **(C):** Gene expression analysis comparing single and pooled culture for markers characterizing all key retinal cell types. *n = 3,* error bars = SEM. Significance assessed by one way ANOVA with Tukey's multiple comparisons test. (B, C) Data presented as average of WT1 and WT2 results.

To enable large‐scale automation and drug testing using retinal organoids, we tested the adaptability of retinal differentiation protocol to 96‐well plates (Fig. 4A). We used for these experiments iPSC lines derived from unaffected individuals and among these, we chose WT1 and WT2 iPSC as they showed similar efficiency and kinetics of differentiation using the 3D retinal protocol described above. Assessment of organoids at day 150 of differentiation indicated that single culture conditions enhanced the ability of wild‐type iPSC to form retinal organoids with RPE (Fig. 4B). In accordance, quantitative RT‐PCR analysis indicated a higher expression of RPE marker (*MITF*; Fig. 4C). No significant differences were observed in the expression of photoreceptor precursor markers (*RCVRN*), cone opsin markers *(OPN1SW, OPN1MW*, and *OPN1LW)* and rod markers (*RHO*), bipolar cell marker (*PKC‐α*), and horizontal, amacrine, and bipolar cell marker (*PROX1*) between organoids maintained all together (pooled) or as single organoids (single, Fig. 4C) at day 150 of differentiation. In contrast, the expression of *AP2‐α,* an amacrine cell marker showed a lower expression in single organoid cultures, whilst the expression of RGC cell markers (*MATH5* and *RBPMS*) was significantly higher in the single organoid cultures (Fig. 4C), thus suggesting that development of these two retinal lineages was affected by single‐cell organoid culture protocol. Transmission electron microscopy analysis showed the presence of photoreceptors at the apical layer of the organoids, containing mitochondria rich inner segments (Supporting Information Fig. S4A,B), connecting cilia, and formation of an outer limiting like membrane. We also observed the presence of photoreceptor outer segments with clear striations inside, indicative of disc membranes. These features were observed in both pooled and single organoid cultures.

### Photoreceptor‐Driven Activity in Retinal Organoid RGCs

**Figure 5:**
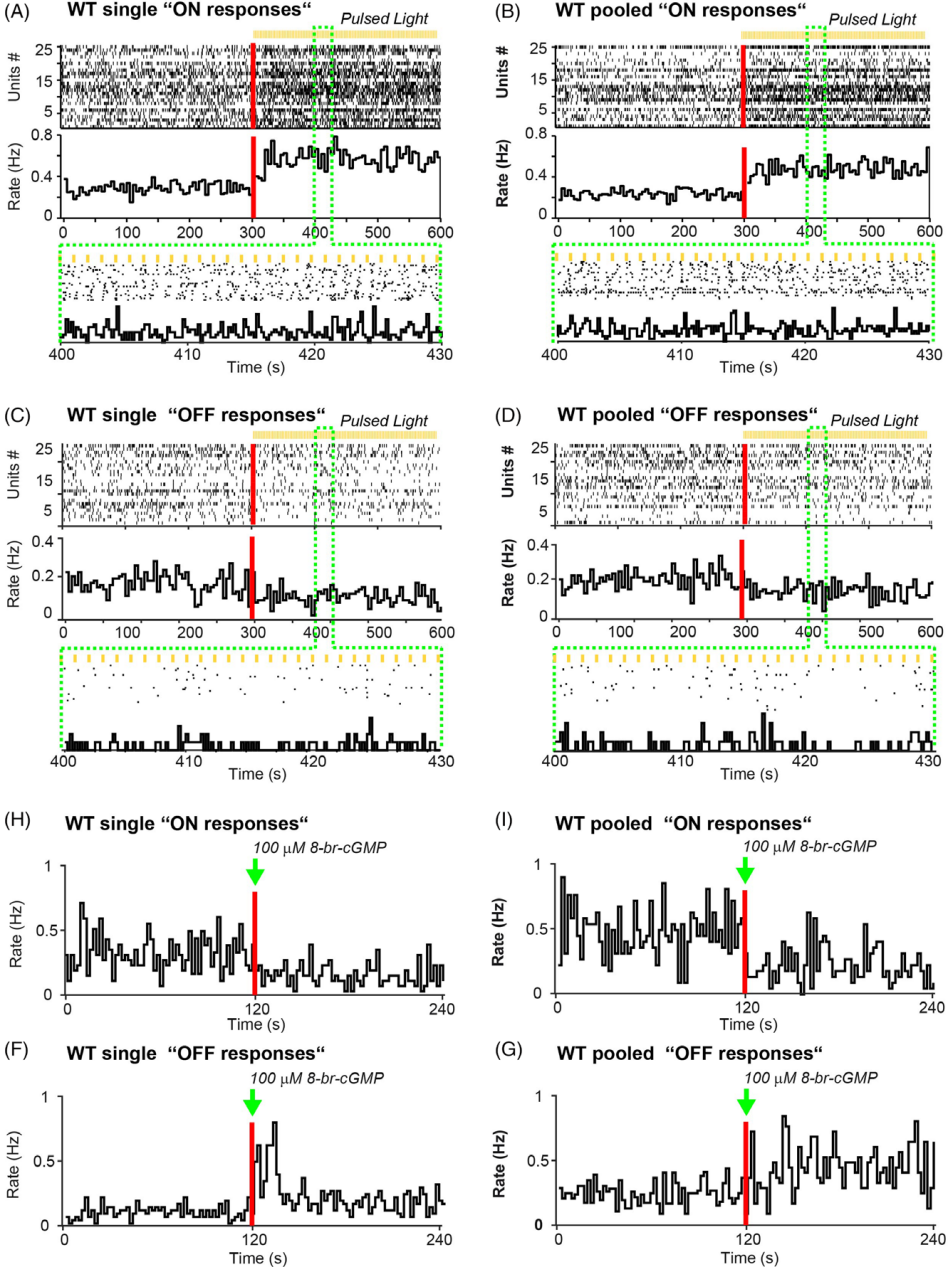
iPSC derived retinal organoids respond to broad white light and cGMP. Spiking activity recorded from presumed RGCs in 3D retinas derived from unaffected (WT) pooled and single organoid cultures at day 150 of differentiation. **(A–D)**: Raster plots (top panels), firing rate histograms (bottom panels, bin size = 5 seconds) and magnification of both (green box, bin size = 0.2 second) with 25 representative RGCs which showed a 25% increase (A,B) or decrease (C,D) in spiking activity in the presence of pulsed light. In the raster plot, each small vertical bar indicates the time stamp of a spike, where each row represents a different RGC. The rate histogram illustrates the number of spikes per defined time window (here 5 seconds) divided by the total number of RGCs. The left half illustrates the activity in control conditions and, separated by the red line, the right half the activity when the 3D retinas are exposed to pulsed light. The green box zooms to a 30 seconds time window during pulsed light with a smaller bin size (0.2 second). **(E–H):** Firing rate histograms (bin size = 2 seconds) of 25 representative RGCs which show either a decrease (E,F) or increase (G,H) in firing in the presence of 8‐br‐cGMP, a membrane permeable analogue of cGMP. The drug was puffed in the recording chamber (final concentration, 100 μM) at the time indicated by the green arrow. The left half illustrates the activity in control conditions.

Presumed RGCs from (WT) pooled and single retinal organoids at day 150 responded either with an overall increase or decrease in spiking activity when exposed to high‐intensity pulsed light (Fig. 5A–D). These responses, however, were too sluggish to allow clear detection of synchronization with the fast light pulses (Fig. 5A,B magnified views). Light responses from 25 representative RGCs (exhibiting at least 25% increase or decrease in spiking activity after the stimulus onset) are illustrated in Figure 5A–D, revealing response patterns similar to those normally seen in immature ON and OFF RGCs in the developing retina (see next paragraph). Quantification of all light‐induced RGC responses (falling within the 25% criterion) showed no significant differences between single or pooled organoids (Mann–Whitney *U* test, Supporting Information Table S3). Interestingly, light had a stronger effect on the spiking activity of organoids derived from WT cultures than in those derived from AMD iPSC line (Supporting Information Fig. S4C–E). To ascertain that the light responses we recorded from organoids are driven by phototransduction in photoreceptors rather than by intrinsically photosensitive RGCs 49, 50, we recorded responses following puffs of cGMP onto the organoids. cGMP is a secondary messenger in the phototransduction cascade that gates Na^+^‐permeable channels in the outer segments of photoreceptors. Puffing 8‐br‐cGMP, a membrane permeable analogue of cGMP, in the recording chamber triggers Na^+^ influx and depolarizes photoreceptors, thus mimicking the dark current. Organoid RGCs responding to the 8‐br‐cGMP puff with decreased spiking activity are thus presumably photoreceptor‐driven ON RGCs (Fig. 5E,F). There was no significant difference between single and pooled ON RGCs in terms of reduced firing rate in response to cGMP puffs (Supporting Information Table S3). We also recorded from cells with increased firing rate following cGMP puffs. These cells presumably are OFF RGCs (Fig. 5G,H). Single OFF RGCs showed a significantly (*p* = .0079) higher activity compared with OFF RGCs from pooled organoids. GABA signaling emerges early during CNS development, it is depolarizing at very early developmental stages and can even induce spiking in RGCs. Later it switches to become the main inhibitory neurotransmitter. Hence, responses to GABA puffs are indicative of emerging functional neural networks. We also recorded changes in firing rate (increase or decrease) following GABA puffs both in single and pooled organoid RGCs (Supporting Information Table S3). Overall, numerous RGCs from single and pooled retinal organoids exhibited clear responses to light, cGMP and GABA stimulation, suggesting rudimental but functional retinal circuitry in both types of organoids.

**Figure 6:**
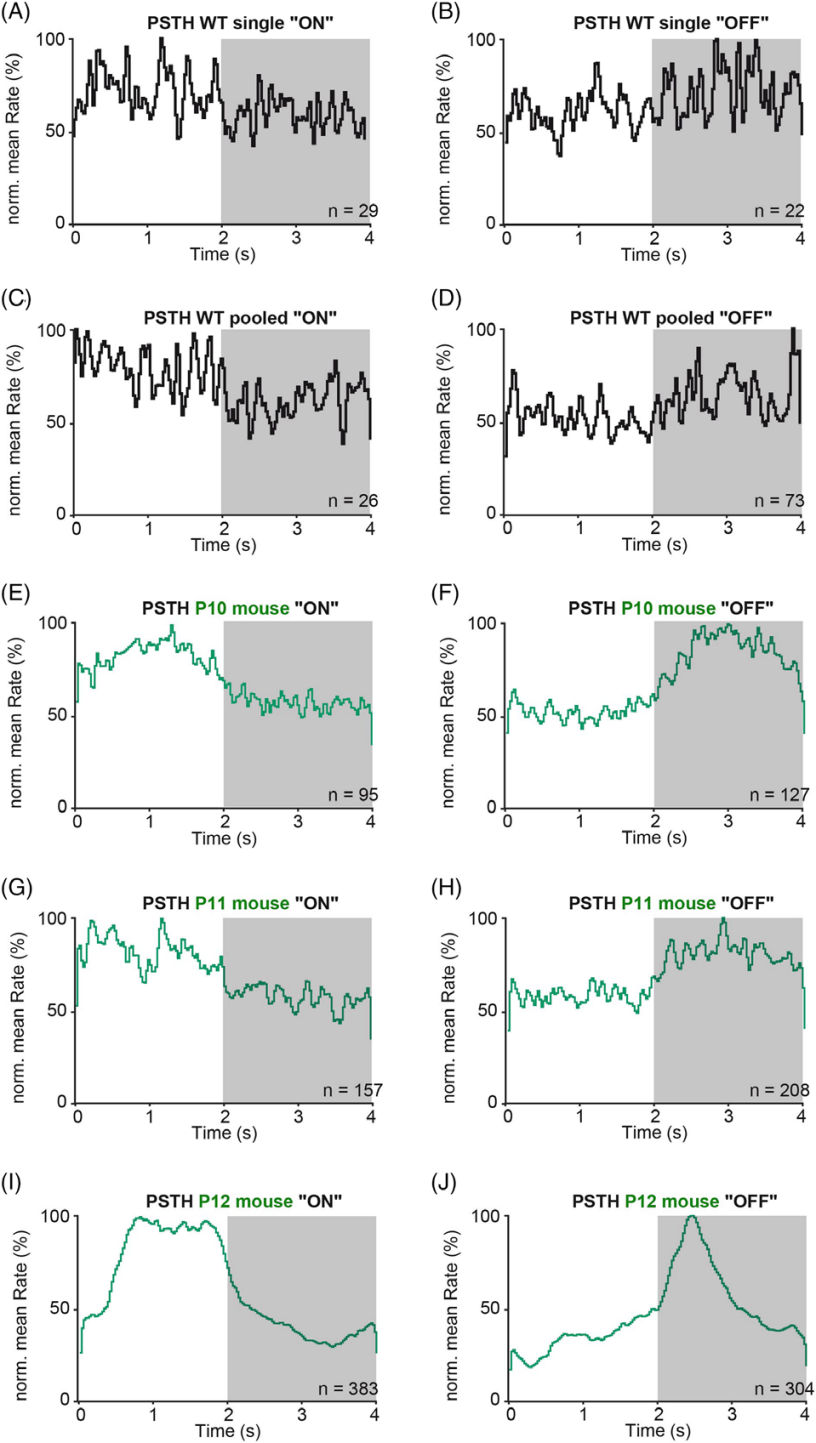
iPSC‐derived retinal organoids show comparable light responses as mice retinas before eye opening under mesopic conditions. Peristimulus time histograms (PSTH) from single and pooled organoid RGCs at day 150 of differentiation (black) and from P10–12 mice retinas (blue). **(A, C):** Presumable ON RGCs from retinal organoids show a slight increase of firing during stimulus onset (0–2 seconds). **(B, D)**: During stimulus offset (2–4 seconds, dark background) the putative OFF RGCs from retinal organoids show slightly more activity. **(E, G, I):** PSTHs of ON P10‐P12 RGCs. **(F, H, J):** PSTHs of OFF P10‐P12 RGCs. The number of RGCs used for PSTH calculation is given in the graph.

The aforementioned light responses from single and pooled organoids were too slow to synchronize with the temporal structure of the fast light pulses. Instead, they exhibited sluggish kinetics reminiscent of immature RGC light responses during the perinatal period or around the time of eye opening 39, 49, 50. Here, we compared the light responses of single and pooled organoids (Fig. 6A–D) with the responses recorded from mouse RGCs at postnatal day (P)10–12 (Fig. 6E–J). P10 is when mouse RGCs become first driven by light originating from the photoreceptor–bipolar cell axis 49, 50, 51, 52, and P12 is the time of eye opening (Fig. 6E–J), roughly corresponding to birth in humans. The neonatal vertebrate retina is characterized by the presence of spontaneous waves of activity sweeping across the RGC layer, disappearing at eye opening in mouse 48, hence it can be challenging to isolate weak nascent light responses within these large barrages of spontaneous bursting activity. Therefore, we used peristimulus time histograms averaged across many stimulus repetitions to average spontaneous bursting out, revealing the presence of weak underlying light‐driven responses. Single and pooled organoid RGCs showed a slight increase in activity either after stimulus onset (Fig. 6A,C) or offset (Fig. 6B,D), reflecting ON and OFF RGCs (it is worth noting that these organoid responses are weaker than those presented in Figure 5 because they were triggered at much weaker light intensities). These responses were weaker than comparable PSTHs from ON (Fig. 6E,G,I) and OFF RGCs (Fig. 6F,H,J) recorded from neonatal retinas, but at least in terms of their sluggish response kinetics, they were reminiscent of responses seen just before eye opening (P10–11; at P12 responses already become much brisker), suggesting that the basic retinal machinery (phototransduction cascade and synaptic circuitry) is present and relatively similar in organoids and in the maturing retina.

### Using Multifactorial Design to Improve Retinal Organoid Generation

**Figure 7:**
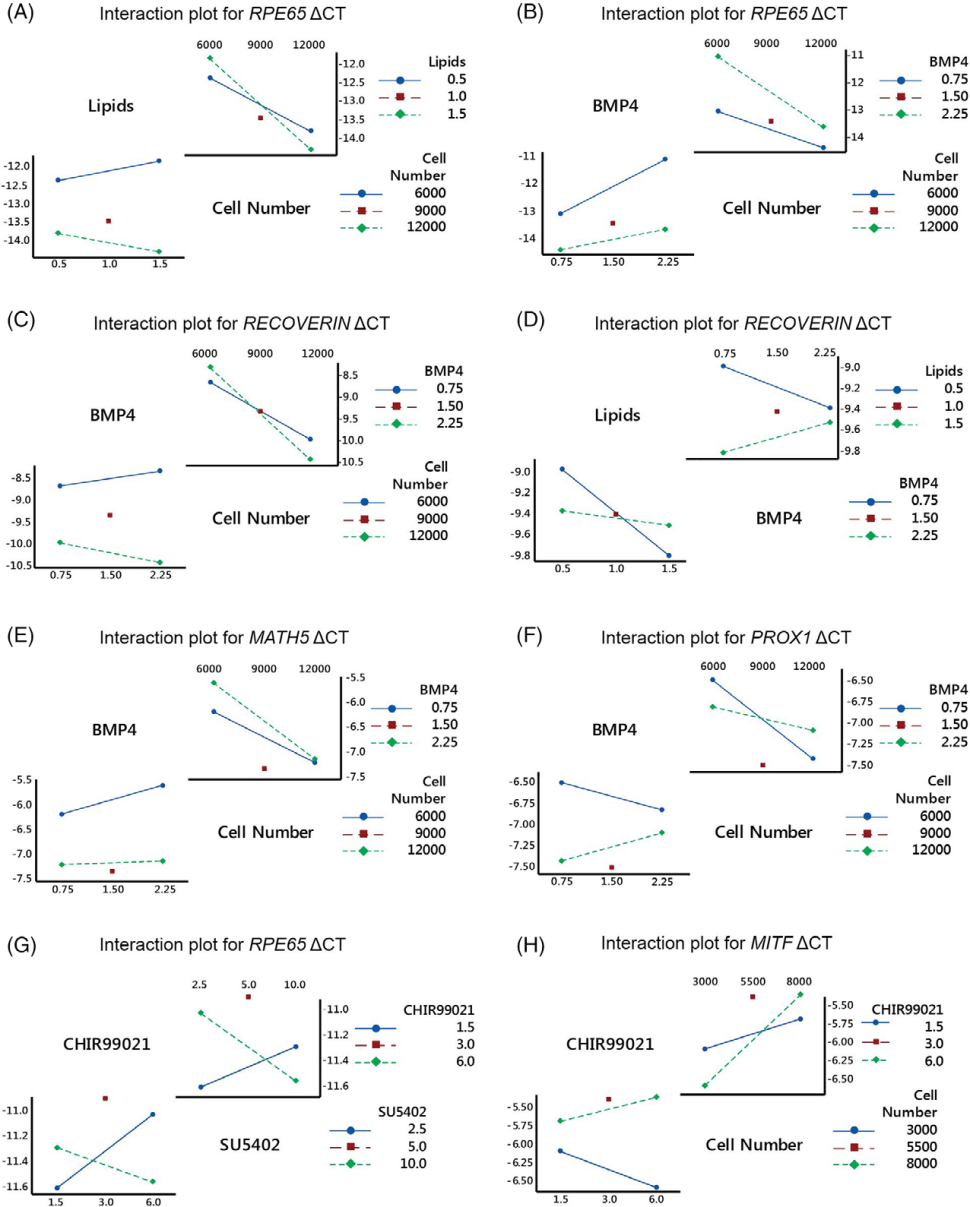
Cell number plays a key role in the generation of retinal organoids from iPSC. **(A–F):** Experimental design 1. (A) Interaction plot for *RPE65*, relationship between lipids and cell number. (B) Interaction plot for *RPE65*, relationship between BMP4 and cell number. (C) Interaction plot for *RCVRN* relationship between BMP4 and cell number. (D) Interaction plot for *RCVRN*, relationship between lipids and BMP4. (E) Interaction plot for *MATH5*, relationship between BMP4 and cell number. (F) Interaction plot for *PROX1*, relationship between BMP4 and cell number. **(G, H):** Experimental design 2: (**G**) interaction plot for *RPE65*, relationship between CHIR99021 and SU5402. (**H**) Interaction plot for *MITF*, relationship between CHIR99021 and cell number. *n = 3* independent replicates.

A two‐level factorial experimental design (Supporting Information Fig. S5) was conducted at day 35 of differentiation in WT2 using a 96‐well format throughout, to determine the key variables in early stage commitment. Eight genes of interest marking various retinal cell phenotypes (*RCVRN, CRX, RPE65, MITF, MATH5, PROX1, VSX2,* and *AP2‐α*), which emerge during differentiation from pluripotent cells, were assessed. Variables were filtered for those that had a statistically significant (*p ≤* .05) influence on the gene expression responses and a magnitude of effect greater than 0.2 cycle threshold (CT; corresponding to a 15% increase or decrease in gene expression). Of the predictor variables evaluated, the cell number was by some margin the most influential on differentiation outcomes across all the responses with a universally statistically significant (*p* ≤ .01 in all cases) and negative correlation with expression (other than with *AP2‐α*, which was positive). Higher concentrations of bone morphogenetic protein 4 (BMP4) had a positive influence on *MATH5* and *RPE65* (*p* ≤ .01 in both cases), whereas higher levels of lipids had a negative, and knockout serum (KSR) a positive effect on *CRX* and *RCVRN* (*p* ≤ .001). These effects are summarized in Supporting Information Table S4A. Several 2‐way interactions were also identified. BMP4 and cell number were nonindependent in their effect on *RPE65*, *RCVRN*, *PROX1*, and *MATH5.* Lipids and cell number were nonindependent in their effect on *RPE65*, whereas lipids and BMP4 were nonindependent in their effect on *RECOVERIN.* The nature of these two‐way interactions are summarized in Fig. 7A–F and Supporting Information Table S4B.

Given the dominance of cell number on the magnitude of effects a further experimental design was evaluated with a lower cell number range and a higher level of BMP4 in combination with two further factors, the canonical/β‐catenin Wnt pathway agonist CHIR99021 and fibroblast growth factor receptor inhibitor SU5402, known inducers of RPE formation 36, 53. The range of delta CT values for each target in experiment 2 was different, particularly for *MATH5* and *VSX2*, which had an extended lower range of outcomes (Supporting Information Table S5A). Once again, cell number was the dominant influence; however, this time the effect was reversed with all responses being positively influenced by a higher cell number (other than *AP2‐α*, which was negative). Expression of *VSX2* and *MATH5* were particularly inhibited at lower cell numbers. This indicates that the optimum cell number is likely close to the 6000–8000 range. Furthermore, a higher level of BMP4 now negatively influenced *MATH5*, *MITF*, and *CRX*, suggesting 2.25 nM had been close to the optimum. Of the newly introduced factors, only CHIR99021 met the criteria for significant effect on responses, with a relatively large magnitude negative influence on *MATH5* and *RCVRN* expression, and a lesser negative effect on *CRX* and *PROX1.* These correlations are summarized in Supporting Information Table S5A. Several two‐way interactions were also identified between CHIR99021 and cell number as shown in Figure 7G,H and Supporting Information Table S5B. Based on this analysis, we determined the optimal conditions for production of retinal organoids containing RPE to be as follows: cell seeding density of 6000–8000 per well, KSR 15%, chemically defined lipid concentrate 0.5%, 1‐thioglycerol 450 μM, BMP4 concentration of 2–2.25 nM, then 3 μM CHIR99021, and 5 μM SU5402.

### iPSC‐Derived Retinal Organoids Respond to Moxifloxacin in a Similar Manner to the Adult Mouse Retina

To assess the response of retinal organoids to known neurotoxicants, we performed proof of principle experiment by exposing day 150 organoids derived from the unaffected control iPSC (WT2) to moxifloxacin. Moxifloxacin is a broad‐spectrum fluoroquinolone antibiotic that is active against both gram‐positive and gram‐negative bacteria and commonly used in the treatment of a range of eye and respiratory infections. The mechanism by which moxifloxacin is retinotoxic is not fully understood; however, a correlation between lipid peroxidation, protein photodamage, and cellular phototoxicity has been observed, indicating that this compound may exert effect in the cellular membranes 54. Doses of 100–500 μg given intracamerally in humans after cataract surgery have not been associated with toxic effects 55, 56. However, in mice 57, a dose‐dependent effect of moxifloxacin has been established with 100 μg/ml and below having no effect on cellular toxicity and cell proliferation, whereas doses of 500 g/ml were reported to result in focal necrosis in the outer nuclear layer (photoreceptor degeneration and loss of photoreceptor inner and outer segments) as well as milder effects on the inner nuclear layer.

Incubation of retinal organoids with 100 μg/ml moxifloxacin led to vacuous apertures to appear in the apical region where photoreceptors reside and the adjacent presumptive inner nuclear layer (Supporting Information Fig. S6). Lactate dehydrogenase (LDH), a cytosolic enzyme that is released when plasma membranes are damaged, revealed a linear dose‐dependent toxicity effect of moxifloxacin (Supporting Information Fig. S6B). Caspase 3, a protease which is involved in executing apoptosis, was expressed in a greater number of cells in retinal organoids exposed to 100 and 300 μg/ml moxifloxacin (Supporting Information Fig. S6C). Gene expression analysis was carried out to identify the affected retinal cell types (Supporting Information Fig. S6D), this showed a significant reduction in the expression of photoreceptor precursor markers (*RCVRN* and *CRX*) and amacrine cell marker (*AP2‐α*) as well as a significant increase in the expression of DNA damage markers *DR5* and *p21.* No changes were observed in the expression of RPE marker (*MITF*; Supporting Information Fig. S6D); however, the expression of bipolar cell marker (*PKC‐α*), horizontal cell marker (*LHX1*), RGC marker (*RBPMS*), and Müller glia cells (*RLBP1*) was increased, most likely reflecting a change in retinal cell subpopulations within these organoids upon the significant decrease of photoreceptor and amacrine cells. Secretome analysis identified a number of differentially regulated proteins (Supporting Information Fig. S6E), notably pigment epithelium‐derived factor (PEDF) and complement component 3 (C3) proteins which are secreted from RPE cells, which not only affirms their presence but also indicates a response to drug treatment. Increases in PEDF and C3 may be a response to inflammatory and stress responses 58, thus increased C3 secretion and deposition may be due to increased number of dead and damaged cells. Macrophage migration inhibitory factor (MIF) was also secreted following treatment, MIF has previously been shown to be released by retinal ganglion cells following neural progenitor cell death 59. Thus, the secretome data may indeed suggest that damage and death of the photoreceptors may be exacerbated by the secreted proteins from other retinal cell layers. Enrichr analysis using the GO biological processes (2017b) database, highlighted protein repair and rhodopsin metabolic processes indicating photoreceptor involvement (Supporting Information Fig. S6F). Together, our results suggest moxifloxacin primarily affects the photoreceptors and amacrine cells of iPSC derived retinal organoids.

## Discussion

Generating reproducible 3D laminated retina from a large range of human pluripotent stem cells with high efficiency and at large scale is crucial for developing ocular disease models and providing a safe test bed for bioactive compounds. In this study, we assessed the ability of five iPSC lines to generate laminated and light‐sensitive retinal organoids using a published protocol 36 which is based on the activation of BMP for a defined time window to enable generation of neural retina followed by a reversible inhibition of GSK3β and FGFR to promote the emergence of RPE. Our data indicate that all iPSC were able to generate laminated retinal organoids with or without RPE. The iPSC‐derived retinal organoids were responsive to light, cGMP and GABA at day 150 of differentiation. Although previous studies showed that transplantation of pluripotent photoreceptor stem cells repairs vision in rodents, only two studies to date have demonstrated the presence of light responses in human pluripotent stem cell derived retinal organoids. Zhong et al. showed that only 2 out of 13 randomly selected photoreceptors responded to light after 6–7 months of differentiation 20, and their photosensitivity was much lower than in normal, adult primate photoreceptors. A more recent study has reported responses to light (530 nm) in 4 out of 25 neurones from 10 organoids between 7 and 9 months of maturation, suggesting the establishment of functional neuronal networks in these structures 66. In the present study, thanks to the very high‐recording yield of our large‐scale, high‐density multielectrode array system, we were able to successfully record light responses in over 1,300 cells (616 cells from single organoids and 710 cells from pooled organoids). From these cells, 229 (~37%) in the single and 179 (~25%) in the pooled conditions responded to light with at least 25% increase in spiking activity. The kinetics of these responses were reminiscent of early light responses recorded from RGCs in the developing mouse retina, suggesting that network connectivity was in reasonably advanced stages of development in these organoids. The intensity of the responses was lower than in the developing retina, possibly due to the relatively small numbers of outer segment disks. Together, our findings suggest that the iPSC‐derived retinal organoids generated in this study contain not only more functionally mature neurones than in previous studies but that maturation also occurred faster than previously reported.

Using statistical DoE methodology, we were able to determine variables critical for the generation of retinal organoids, which indicated starting cell number, BMP4, KSR, and lipids to be the main constituents in successful formation of neural retina and CHIR99021, SU5402, and starting cell number in RPE generation. The optimizations performed in this study with the WT iPSC lines could be applied to any potential iPSC line to aid in the generation of laminated retinal organoids. We successfully transferred this differentiation protocol to 96‐well plates for the whole duration of the experiment, allowing for the scalability, and automation required for toxicology and pharmacology studies.

Variabilities in the ability of iPSC lines to respond to differentiation protocols are now widely reported in the literature and thought to be influenced by many factors including perturbed signaling pathways and chromatin regulators 60, 61, 62. Methylation status also plays a dominant role on differentiation propensity 63. Our own work and that of others have shown that differences in the activity of key signaling pathways such as TGFβ and BMP signaling can have a large impact on the ability of human iPSC to respond to differentiation protocols 64, 65. A growing number of studies to date have used human iPSC lines to generate retinal organoids via different protocols, showing that those do indeed efficiently recapitulate retinogenesis 17, 20, 66; however, the line to line variability has not been fully addressed to date. Our study compared five different iPSC lines from unaffected subjects and those with AMD and inherited RP and showed that significant differences exist with reference to kinetics of differentiation and efficiency of generating retinal organoids. Some of the iPSC lines were able to generate organoids with apically positioned photoreceptor progenitors and basally located ganglion and/or amacrine cells as early as day 35 of differentiation; however, in some others, these were just beginning to develop by day 60 of differentiation, thus indicating different kinetics which was closely paralleled by gene expression analysis. Some of the cell lines that were slow in their early differentiation steps caught up by day 150 with the fastest cell lines in terms of overall differentiation efficiency; nonetheless, the gene expression analysis indicated that the fastest iPSC lines had the highest expression of cones, rods, horizontal, amacrine, and bipolar cells which most likely reflects a more advanced differentiation stage. The large majority of retinal organoids generated by the iPSC lines did not contain any pigmented RPE patches and even for the lines that did generate retinal organoids with RPE this comprised a small fraction of total organoids (maximum 9%). In addition to retinal organoids, human iPSC lines did also generate pigmented hollow RPE spheres, which have not been described to date in the literature. Again, we observed a large variance with some iPSC lines generating as much as 61% RPE spheres and some others none at all. Overall, the ability to give rise to RPE either as part of retinal organoid or on its own was variable between the lines and corroborated closely by the expression of RPE marker (*MITF*) and RPE and Müller glia cell marker (*RLBP1*). Together, our data highlight the variability of iPSC lines to generate retinal organoids and the need for fully characterizing a large number of iPSC lines carefully to select those that can efficiently differentiate to laminated retinal organoids.

Starting cell number of retinal organoids has been described as an influential factor for the success of retinal organoid generation 31. Previous studies have used ranges between 1,000–9,000 32 and 9,000–12,000 14, 36, reporting that lower seeding numbers produced fewer distinct optic vesicles, higher cell numbers develop necrotic cores by day 10 32, suggesting 3,000 to be the optimum number. To assess the role of seeding cell number together with other important growth factors and small molecules used in the differentiation protocol, we performed statistical DoE. We tested cell seeding numbers between 3,000 and 12,000 per aggregate, and found that these numbers played the biggest single effect on the measured outcomes, with a predicted optimal range between 6,000 and 8,000 cells. BMP4 supplementation also had a significant role on gene expression, initially in Design 1, higher BMP4 concentrations were more favorable for RPE and RGCs development; however, increasing the concentration further in Design 2 appeared detrimental, thus indicating 2–2.25 nM range to be optimal. These results corroborate previously published data indicating that high‐BMP4 concentrations lead to reduced differentiation of photoreceptors 63. Removing 32 or reducing KSR 14 has been shown to inhibit the formation of optic vesicles, whereas higher concentrations may lead to caudalization. In our design, 15% proved to be the optimal concentration, promoting development of photoreceptor precursors as revealed by CRX and RCVRN expression. The process control highlighted that only AP2‐α (amacrine cell maker) was elevated at higher seeding density, while MATH5 expression increased at lower densities. Likewise AP2‐α was decreased in single organoid cultures, whereas MATH5 was increased, indicating that amacrine cells perhaps benefit from lower nutrient availability, while RGCs have a greater need for nutrients based on media availability per cell, thus highlighting a possible link between nutrient availability and cell fate. This corroborates previously published data showing that nutrient depletion causes a block in proliferation and differentiation of retinal progenitors in the ciliary margin zone 64. A major advantage of the statistical design was the ability to assess compounding effects, with lower cell numbers and higher concentrations of BMP4 increasing the gene expression of photoreceptors, RGCs and RPE. However, the lower cell numbers and higher concentrations of BMP4 had the converse effect on amacrine cells. These insights highlight that optimal growth conditions would be a compromise between the requirements of the many different cell types present in the retinal organoids.

To enable automation of iPSC differentiation and to test scalability, we maintained half of the retinal organoids in the U bottomed 96‐well plates (one retinal organoid per well) and compared those to organoids maintained in 6‐well plates using an identical nutrient regime in both populations. Single organoid conditions enhanced generation of retinal organoids with RPE, encouraged better RGC differentiation and response to cGMP. However, the expression of all other retinal cell markers, the efficiency of retinal organoid generation and light responses were similar between the two conditions, thus validating the 96‐well plate protocol for prolonged (up to 150 days) differentiation to retinal organoids.

To validate the application of iPSC derived retinal organoids, we went on to compare their response to moxifloxacin, an antibiotic that is commonly used to treat eye infections and which has been shown to affect the outer and inner nuclear layer in mouse retina, at a dose of 500 μg/ml 57. When applied to iPSC derived retinal organoids moxifloxacin had a clear dose‐dependent effect, shown by LDH, gene expression and secretome data, with higher dosages leading to disintegration of the organoids (data not shown), resulting in reduced RNA transcript recovery. In our experiments, 300 μg/ml and above had the greatest effect on gene expression, whereas in the mouse doses of 500 μg/ resulted in degeneration of photoreceptors and mild disruption of the inner nuclear layer 57. These differences in sensitivity to moxifloxacin may be because iPSC‐derived retinal organoids are more similar to fetal retina in terms of cellular composition and transcription profiles 67. Despite these differences, our gene expression and immunocytochemical analysis identified the photoreceptors and amacrine cells to be the key affected cell types, indicating a similar response to the in vivo adult mouse retina 57.

## Conclusion

In summary, our data show a significant variability between different iPSC lines in their overall efficiency to generate light responsive retinal organoids, in the kinetics of differentiation and ability to generate RPE either as part of organoids or by itself; thus, it is important to carefully select iPSC lines that respond robustly to the differentiation process and can be used for compounds screening and clinical trials. We also show that the differentiation process is highly dependent on nutrient availability. Through statistical design experiment, we have determined the optimal application of key growth factors and small molecules as well as cell seeding density to generate light and drug responsive laminated retinal organoids in a format that allows scalability and automation.

## Author Contributions

D.H. and G.H.: experimental design, execution and manuscript preparation, data analysis; B.D. and A.P.: performed experiments, data analysis; L.Z., M.Y., and S.B.: performed experiments; P.H., M.S., M.U., S.K., D.S., and L.A.: fundraising and experimental design; M.N.: fundraising; R.T.: experimental design, data analysis and manuscript preparation; A.T.: experimental design and data analysis; E.S. and M.L.: experimental design, data analysis, fund raising and manuscript preparation.

## Disclosure of Potential Conflicts of Interest

M.U. declared employment with Novartis Pharma AG. S.K. declared employment with F. Hoffmann‐La Roche Ltd. D.S. declared advisory role with Alcon and research funding from Bayer. M.N. declared employment and stock ownership with Newcells Biotech Ltd. The other authors indicated no potential conflict of interest.

## Supporting information


**Figure S1.** Brightfield images of organoids. Example images of organoids cultured in pooled and single conditions from each cell line tested. Scale bar = 500 μM.

**Figure S2.** Relative organoid size during the differentiation process. Error bars = SEM. Significance assessed by one way ANOVA with Tukey's multiple comparisons test.

**Figure S3.** Schematic chart showing the development of iPSC derived retinal organoids and RPE spheres at day 60 of differentiation. Abbreviations: RPE, RPE spheres; NR, retinal organoids with neural retina (no RPE), NR+ RPE, retinal organoids with RPE; undefined, organoids that did not contain any neural retina or RPE cells, *n = 3.*

**Figure S4.** TEM and electrophysiological analysis. **(A):** Transmission electron micrographs of single and **(B)** pooled retinal organoids at day 150. Abbreviations: OLM, outer limiting membrane; IS, inner segment; PC, primary cilium; M, mitochondria; OS, outer segment. Scale bar far left = 2 μm, scale bar middle and right = 500 nm. **(C):** 25 representative cells from a diseased line (AMD) cultured with the “single” protocol **(A)** showed less increased firing rate than WT cells **(D)** cultured with the identical “single” protocol after they were exposed to strong pulsed light. **(E):** Quantification of the different firing rates between AMD single and WT single. The WT single cells showed a significant stronger increase in their firing rate after pulsed light exposure than AMD single cells (*p < .034; Mann–Whitney *U* test; *N = 151* for AMD single and *N = 145* for WT single).

**Figure S5.** Factorial experimental design. **(A):** Table showing design of factorial experiment 1. **(B):** Table showing design of factorial experiment 2. **(C):** Chart showing overlapping coverage of cell number and BMP4 between the two experiments.

**Figure S6.** Response of iPSC‐derived‐retinal organoids to moxifloxacin treatment. **(A):** Hematoxylin and eosin staining of retinal organoids, left = untreated control and right = Moxifloxacin 100 μg/ml. Red asterisk = disorganization and gaps in laminated structure (Scale bar = 100 μm; *n = 3*). **(B):** LDH tests of retinal organoids treated with Moxifloxacin 50–1000 μg/ml (*n = 3*). **(C):** Caspase 3 (Red) expression in control untreated retinal organoids (left), 100 μg/ml moxifloxacin (middle), and 300 μg/ml (right; scale bar = 50 μm). **(D):** Gene expression of key retinal cell markers*, n = 3,* error bars = SEM. Significance assessed by one way ANOVA with Tukey's multiple comparisons test. **(E):** Heatmap showing clustering of control and 100 μg/ml moxifloxacin treated retinal organoids. **(F):** Enrichr analysis of top 16 upregulated proteins.

Table S1. The DNA sequence of oligonucleotides used in the qRT‐PCR analysis.

Table S2. Summary of antibodies used in this study.

Table S3. Mann–Whitney *U* test on spiking activity.

Table S4. **(A):** Table showing significant single interactions on gene expression for design 1. **(B):** Table showing two way interactions for design 1.

Table S5. **(A):** Table showing significant single interactions on gene expression for design 2. **(B):** Table showing two way interactions for design 2.
