# Supplementary figures and images for: Human‐Induced Pluripotent Stem Cells Generate Light Responsive Retinal Organoids with Variable and Nutrient‐Dependent Efficiency
Source: Stem Cells. 2018 Aug 13;36(10):1535–51. doi: 10.1002/stem.2883 (PMC6392112; doi:10.1002/stem.2883)

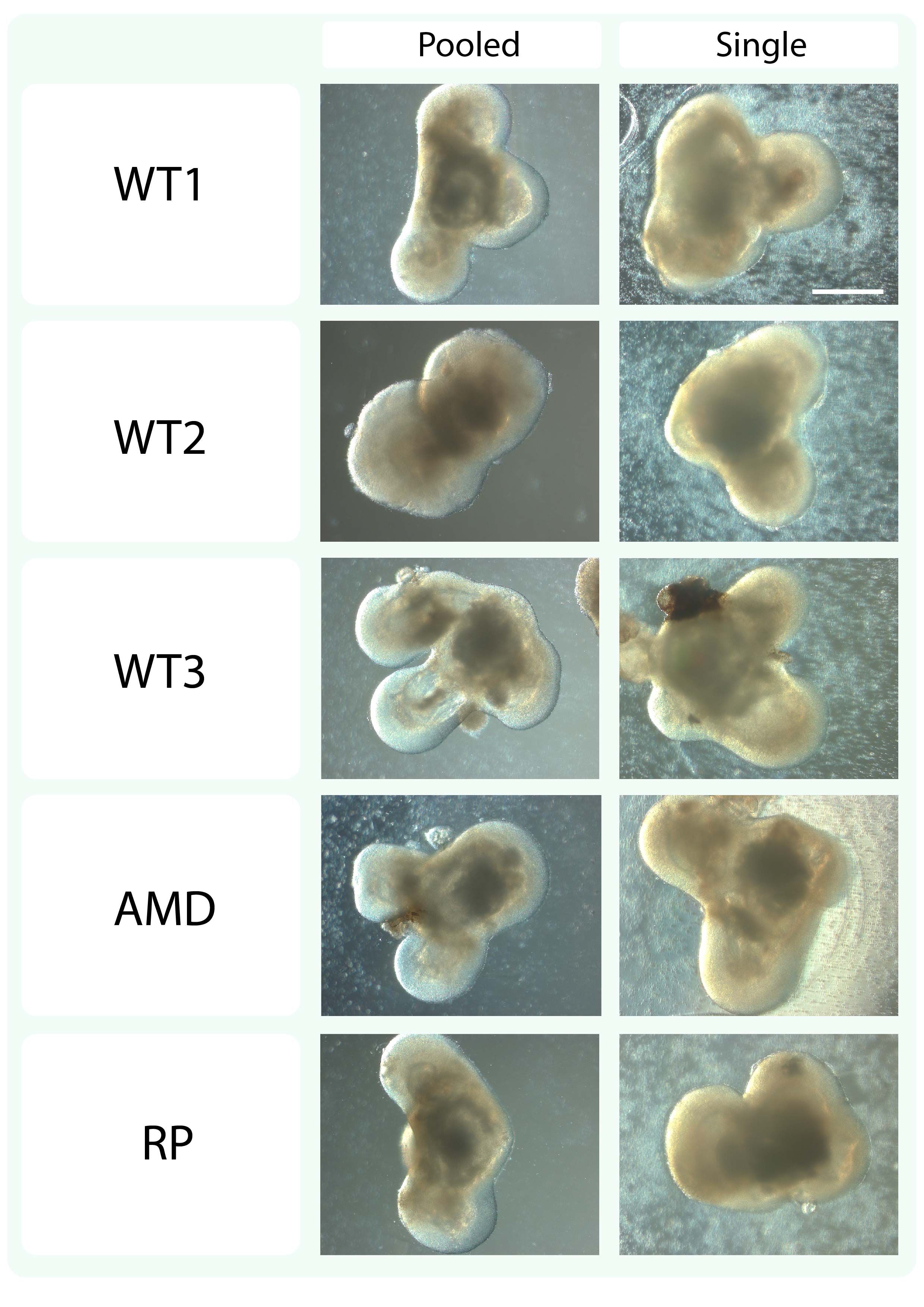

Supplement: Supplementary file 1 — Figure S1. Brightfield images of organoids. Example images of organoids cultured in pooled and single conditions from each cell line tested. Scale bar = 500 μM. [file STEM-36-1535-s003.jpg]

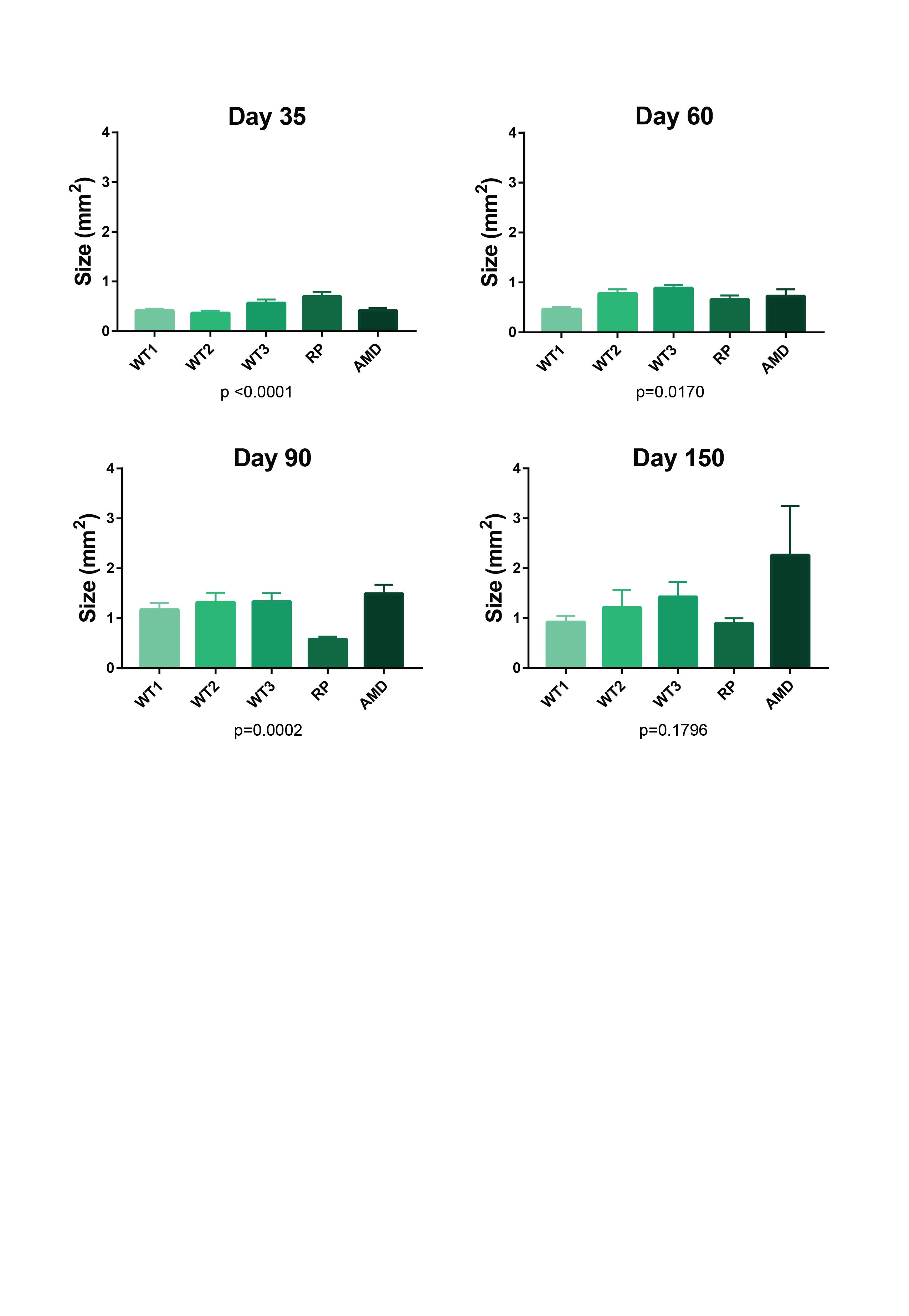

Supplement: Supplementary file 2 — Figure S2. Relative organoid size during the differentiation process. Error bars = SEM. Significance assessed by one way ANOVA with Tukey's multiple comparisons test. [file STEM-36-1535-s004.jpg]

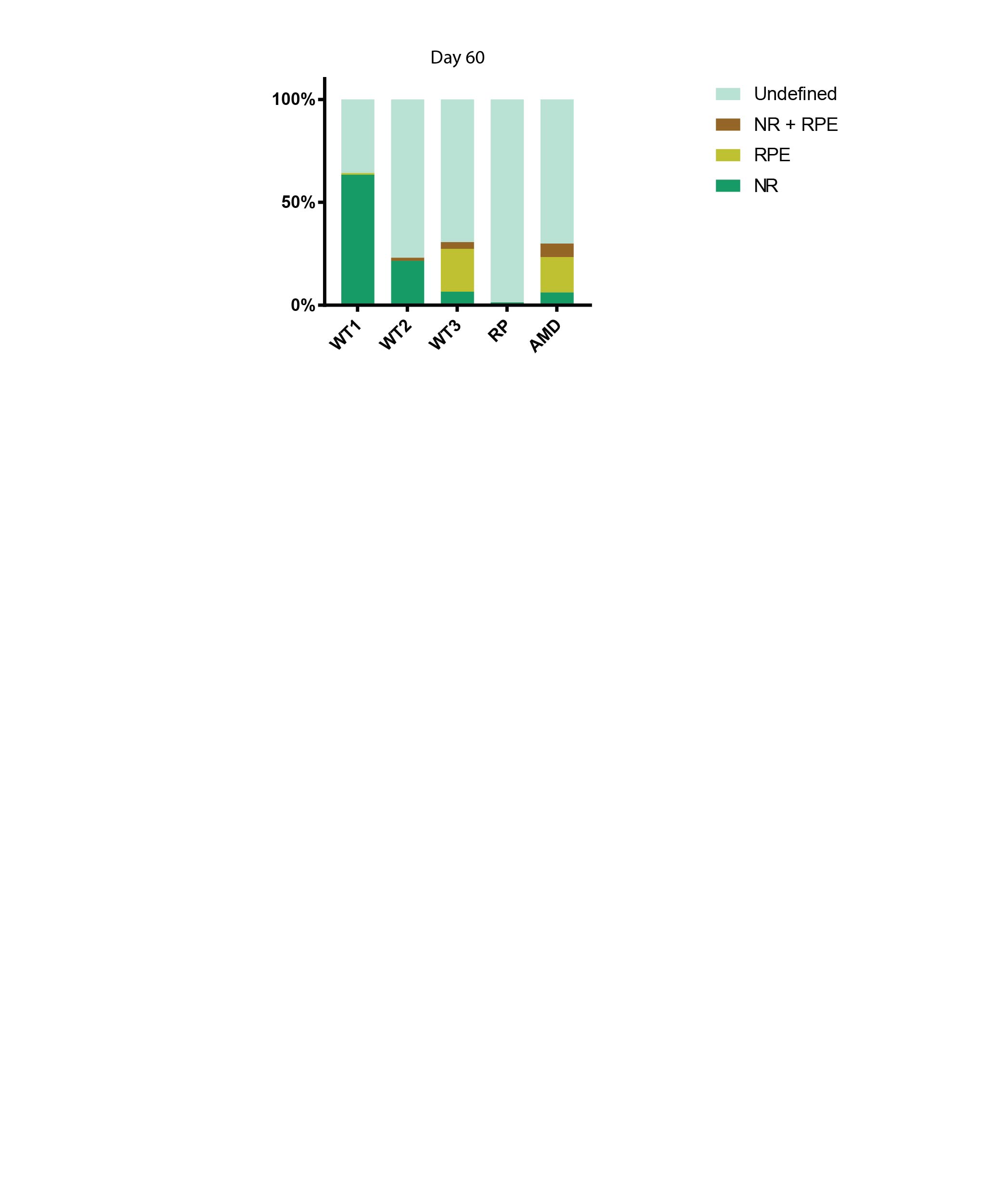

Supplement: Supplementary file 3 — Figure S3. Schematic chart showing the development of iPSC derived retinal organoids and RPE spheres at day 60 of differentiation. Abbreviations: RPE, RPE spheres; NR, retinal organoids with neural retina (no RPE), NR+ RPE, retinal organoids with RPE; undefined, organoids that did not contain any neural retina or RPE cells, n = 3. [file STEM-36-1535-s005.jpg]

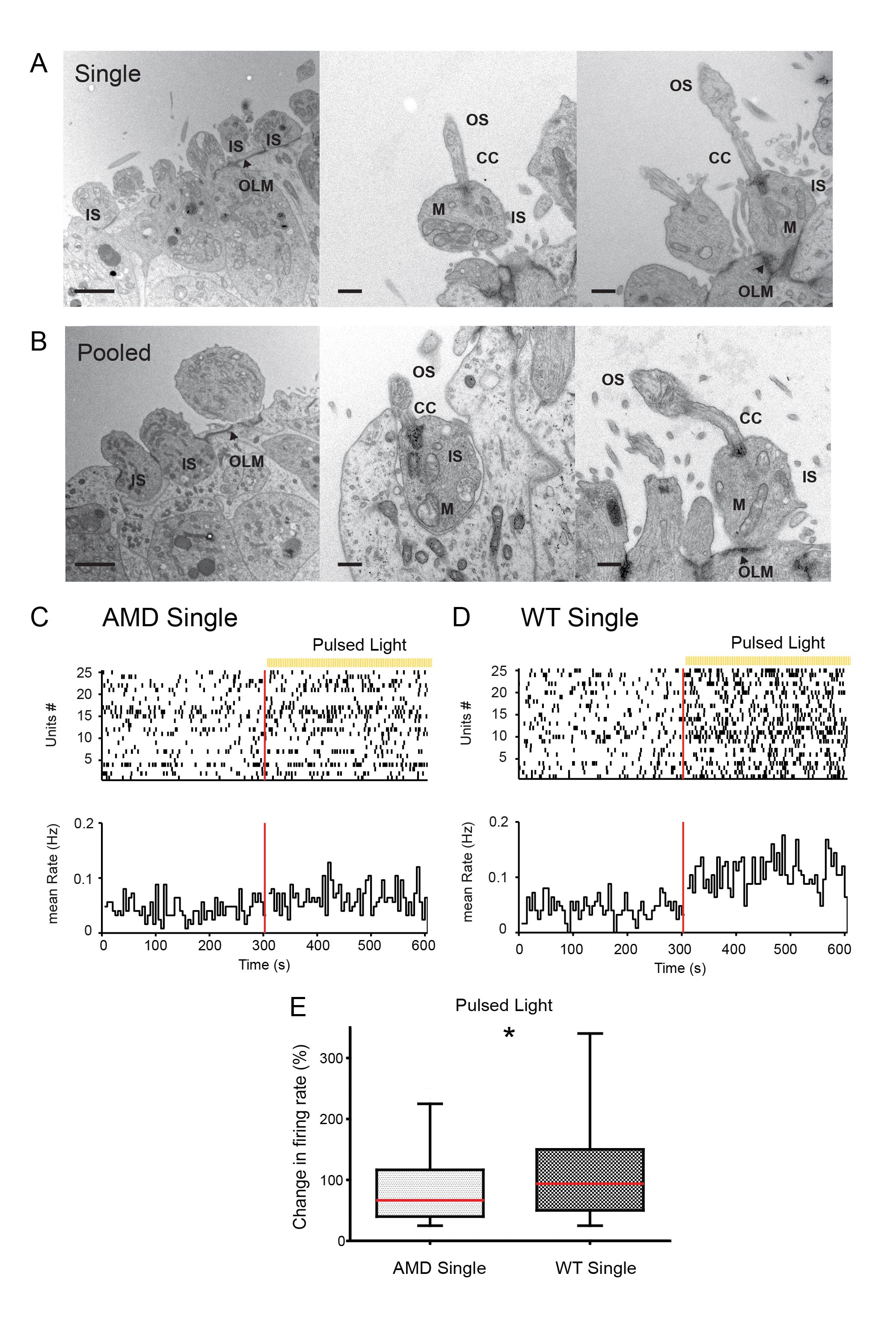

Supplement: Supplementary file 4 — Figure S4. TEM and electrophysiological analysis. (A): Transmission electron micrographs of single and (B) pooled retinal organoids at day 150. Abbreviations: OLM, outer limiting membrane; IS, inner segment; PC, primary cilium; M, mitochondria; OS, outer segment. Scale bar far left = 2 μm, scale bar middle and right = 500 nm. (C): 25 representative cells from a diseased line (AMD) cultured with the “single” protocol (A) showed less increased firing rate than WT cells (D) cultured with the identical “single” protocol after they were exposed to strong pulsed light. (E): Quantification of the different firing rates between AMD single and WT single. The WT single cells showed a significant stronger increase in their firing rate after pulsed light exposure than AMD single cells (*p < .034; Mann–Whitney U test; N = 151 for AMD single and N = 145 for WT single). [file STEM-36-1535-s006.jpg]

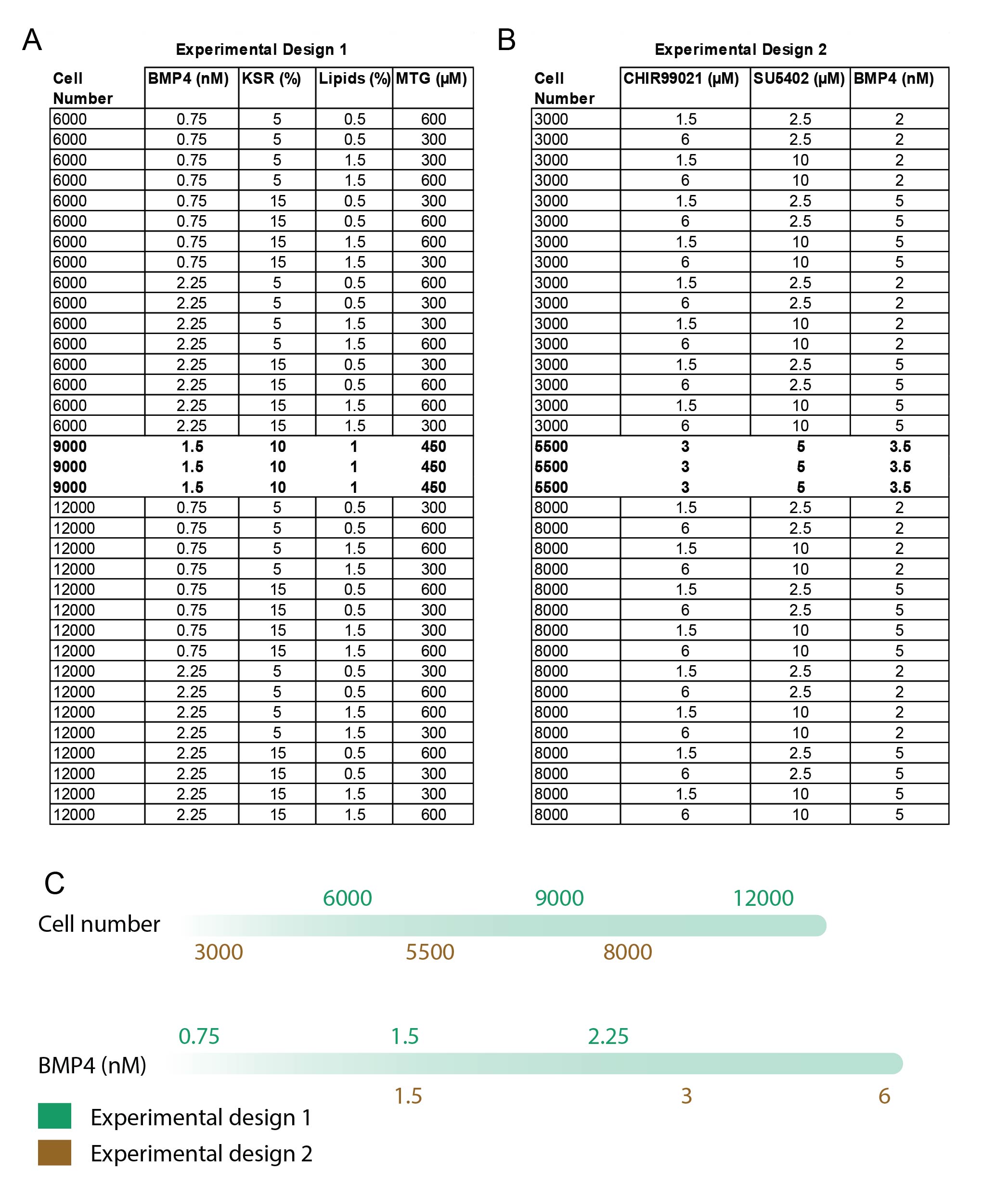

Supplement: Supplementary file 5 — Figure S5. Factorial experimental design. (A): Table showing design of factorial experiment 1. (B): Table showing design of factorial experiment 2. (C): Chart showing overlapping coverage of cell number and BMP4 between the two experiments. [file STEM-36-1535-s007.jpg]

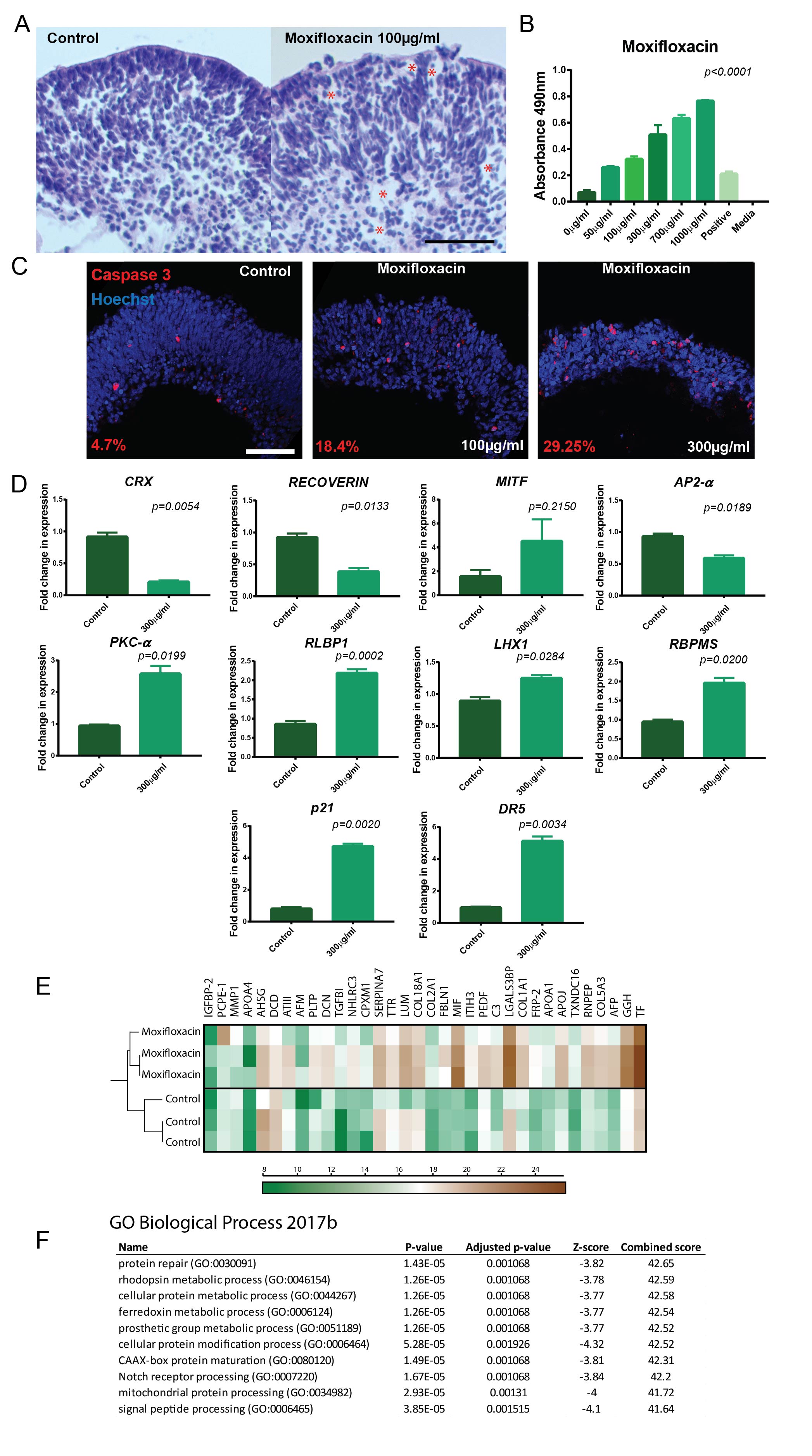

Supplement: Supplementary file 6 — Figure S6. Response of iPSC‐derived‐retinal organoids to moxifloxacin treatment. (A): Hematoxylin and eosin staining of retinal organoids, left = untreated control and right = Moxifloxacin 100 μg/ml. Red asterisk = disorganization and gaps in laminated structure (Scale bar = 100 μm; n = 3). (B): LDH tests of retinal organoids treated with Moxifloxacin 50–1000 μg/ml (n = 3). (C): Caspase 3 (Red) expression in control untreated retinal organoids (left), 100 μg/ml moxifloxacin (middle), and 300 μg/ml (right; scale bar = 50 μm). (D): Gene expression of key retinal cell markers, n = 3, error bars = SEM. Significance assessed by one way ANOVA with Tukey's multiple comparisons test. (E): Heatmap showing clustering of control and 100 μg/ml moxifloxacin treated retinal organoids. (F): Enrichr analysis of top 16 upregulated proteins. [file STEM-36-1535-s008.jpg]
