# Supplementary material for: Human‐Induced Pluripotent Stem Cells Generate Light Responsive Retinal Organoids with Variable and Nutrient‐Dependent Efficiency
Source: Stem Cells. 2018 Aug 13;36(10):1535–51. doi: 10.1002/stem.2883 (PMC6392112; doi:10.1002/stem.2883)
Supplement: Supplementary file 7 — Table S1. The DNA sequence of oligonucleotides used in the qRT‐PCR analysis. [file STEM-36-1535-s009.docx]

| **Gene** | **Direction** | **Sequence** |
| --- | --- | --- |
| *AP2-α* | Forward | GTTACCCTGCTCACATCACTAG |
|  | Reverse | TCTTGTCACTTGCTCATTGGG |
| *MATH5* | Forward | CCCTAAATTTGGGCAAGTGAAGA |
|  | Reverse | CAAAGCAACTCACGTGCAATC |
| *PROX1* | Forward | TGACTTTGAGGTTCCAGAGAGA |
|  | Reverse | CTCTTGTAGGCAGTTCGGGG |
| *CRX* | Forward | GTGAGGAGGTGGCTCTGAAG |
|  | Reverse | CTGCTGTTTCTGCTGCTGTC |
| *RECOVERIN* | Forward | TTCAAGGAGTACGTCATCGCC |
|  | Reverse | GATGGTCCCGTTACCGTCC |
| *VSX2* | Forward | GGCGACACAGGACAATCTTTA |
|  | Reverse | TTCCGGCAGCTCCGTTTTC |
| *GAPDH* | Forward | TGCACCACCAACTGCTTAGC |
|  | Reverse | GGCATGGACTGTGGTCATGAG |
| *RPE65* | Forward | GCCCAGGAGCAGGACAAAAG |
|  | Reverse | GCGCATCTGCAAGTTAAAACCA |
| *RBPMS* | Forward | TGACAGTCGCTCAGAAGCAG |
|  | Reverse | TCACACACCTGGGACATAGT |
| *RLBP1* | Forward | GGCAGGGAACAACCAAGACT |
|  | Reverse | AGTCAGGGCCAAGTTGTGAC |
| *LHX1* | Forward | CGCGTCATTCAGGTCTGGTT |
|  | Reverse | AAGGGTAGGTCCACTGGTGT |
| *RHODOPSIN* | Forward | GGTCCAAAGACACCTGATGG |
|  | Reverse | TGTTGGCAACCCACTAATGA |
| *NRL* | Forward | GGGCTGAGTCCTGAAGAGG |
|  | Reverse | TTTAGCTCCCGCACAGACAT |
| *PKC- α* | Forward | AGAGGGACGTGAGAGAGCAT |
|  | Reverse | CCTTTGCCACACACACTTTGGG |

**Table S1**
