# Supplementary material for: Human‐Induced Pluripotent Stem Cells Generate Light Responsive Retinal Organoids with Variable and Nutrient‐Dependent Efficiency
Source: Stem Cells. 2018 Aug 13;36(10):1535–51. doi: 10.1002/stem.2883 (PMC6392112; doi:10.1002/stem.2883)
Supplement: Supplementary file 8 — Table S2. Summary of antibodies used in this study. [file STEM-36-1535-s010.docx]

| **Antibody** | **Immunogen** | **Host and clonality** | **Dilution** | **Supplier, Cat. No** |
| --- | --- | --- | --- | --- |
| ARL13B | Synthetic peptide corresponding to a region within the internal sequence amino acids 251-300 of Human ARL13B | Rabbit, polyclonal | 1:500 | Abcam, ab83879 |
| Basson, clone SAP7F407 | Recombinant rat Bassoon | Mouse, monoclonal | 1:100 | StressGen, VAM-PS003 |
| CRX, clone 4G11 | CRX partial recombinant protein with GST tag | Mouse, monoclonal | 1:200 | Abnova, H00001406-M02 |
| CRALBP, clone B2 | Human recombinant CRALBP | Mouse, monoclonal | 1:100 | GeneTex, GTX15051 |
| HuC/HuD, clone 16A11 | Human HuC/HuD neuronal protein | Mouse, monoclonal | 1:200 | Invitrogen, A21271 |
| Opsin, clone RET-P1 | Rat retinal membranes | Mouse, monoclonal | 1:200 | Sigma-Aldrich, O4886 |
| Opsin blue | Recombinant human blue opsin | Rabbit, polyclonal | 1:200 | Millipore, ab5407 |
| Opsin red/green | Recombinant human red/green opsin | Rabbit, polyclonal | 1:200 | Millipore, ab5405 |
| PKCα | Human PKCα aa. 270-427 Recombinant Protein | Mouse, monoclonal | 1:200 | BD Transduction Laboratories, 610107 |
| Prox1 | Synthetic peptide from the C-terminus of mouse Prox1 | Rabbit, polyclonal | 1:1500 | Millipore, ab5475 |
| Recoverin | Recombinant human recoverin | Rabbit, polyclonal | 1:1000 | Millipore, ab5585 |
| Syntaxin, clone HPC-1 | Synaptosomal plasma-membrane fraction from adult rat hippocampus | Mouse, monoclonal | 1:200 | Sigma-Aldrich, S0664 |
| VGLUT1 | Synthetic peptide from rat VGLUT1 protein | Guinea pig, polyclonal | 1:1500 | Millipore, ab5905 |
| VSX2 | Visual system homeobox 2 recombinant protein epitope signature tag (PrEST) | Rabbit, polyclonal | 1:50 | Sigma-Aldrich, HPA003436 |
| Caspase 3 | Synthetic peptide corresponding to Human Caspase-3 aa 150-250 | Rabbit, polyclonal | 1:200 | Abcam, ab13847 |

**Table S2**
