# Supplementary material for: Human‐Induced Pluripotent Stem Cells Generate Light Responsive Retinal Organoids with Variable and Nutrient‐Dependent Efficiency
Source: Stem Cells. 2018 Aug 13;36(10):1535–51. doi: 10.1002/stem.2883 (PMC6392112; doi:10.1002/stem.2883)
Supplement: Supplementary file 9 — Table S3. Mann–Whitney U test on spiking activity. [file STEM-36-1535-s011.docx]

|  | **units with more than 25% decreased firing rate** | | | | | |
| --- | --- | --- | --- | --- | --- | --- |
|  | **Pulsed Light** | | **cGMP** | | **GABA** | |
|  | WT single | | WT single | | WT single | |
|  | vs | | vs | | vs | |
|  | WT pooled | | WT pooled | | WT pooled | |
|  |  | |  | |  | |
| Mann Whitney test |  | |  | |  | |
| P value | 0.4659 | | 0.4323 | | 0.131 | |
| Exact or approximate P value? | Gaussian Approximation | | Gaussian Approximation | | Gaussian Approximation | |
| P value summary | ns | | ns | | ns | |
| Are medians signif. different? (P < 0.05) | No | | No | | No | |
| One- or two-tailed P value? | Two-tailed | | Two-tailed | | Two-tailed | |
| Sum of ranks in column F,G | 689 , 637 | | 15929 , 15698 | | 17153 , 13973 | |
| Mann-Whitney U | 283 | | 7414 | | 6713 |  |
|  |  |  |  |  |  |  |
|  | WT single | WT pooled | WT single | WT pooled | WT single | WT pooled |
| Number of values | 28 | 23 | 130 | 121 | 144 | 105 |
|  |  |  |  |  |  |  |
| Minimum | -66.55 | -58.62 | -100 | -100 | -85.71 | -100 |
| 25% Percentile | -46.47 | -46.38 | -58.33 | -50 | -50 | -48.02 |
| Median | -33.1 | -31.82 | -40 | -38.46 | -40.59 | -37.5 |
| 75% Percentile | -30.58 | -27.08 | -31.83 | -31.08 | -33.33 | -30 |
| Maximum | -25 | -25 | -25 | -25 | -25.64 | -25 |
|  |  |  |  |  |  |  |
| Mean | -38.14 | -37.37 | -46.34 | -44.11 | -43.29 | -43.47 |
| Std. Deviation | 11.35 | 11.67 | 19.41 | 18.16 | 12.97 | 19.01 |
| Std. Error | 2.144 | 2.434 | 1.702 | 1.651 | 1.081 | 1.856 |
|  |  |  |  |  |  |  |
| Lower 95% CI of mean | -42.54 | -42.42 | -49.71 | -47.38 | -45.43 | -47.15 |
| Upper 95% CI of mean | -33.74 | -32.33 | -42.98 | -40.84 | -41.16 | -39.79 |
|  |  |  |  |  |  |  |
| Sum | -1068 | -859.6 | -6025 | -5338 | -6234 | -4564 |
|  |  |  |  |  |  |  |
|  |  |  |  |  |  |  |
|  | **units with more than 25% increased firing rate** | | | | | |
|  | **Pulsed Light** | | **cGMP** | | **GABA** | |
|  | WT single | | WT single | | WT single | |
|  | vs | | vs | | vs | |
|  | WT pooled | | WT pooled | | WT pooled | |
|  |  | |  | |  | |
| Mann Whitney test |  | |  | |  | |
| P value | 0.3168 | | 0.0079 | | 0.3474 | |
| Exact or approximate P value? | Gaussian Approximation | | Gaussian Approximation | | Gaussian Approximation | |
| P value summary | ns | | ** | | ns | |
| Are medians signif. different? (P < 0.05) | No | | Yes | | No | |
| One- or two-tailed P value? | Two-tailed | | Two-tailed | | Two-tailed | |
| Sum of ranks in column F,G | 47657 , 34559 | | 38955 , 33816 | | 36955 , 44049 | |
| Mann-Whitney U | 18983 | | 15288 | | 18849 | |
|  |  |  |  |  |  |  |
|  | WT single | WT pooled | WT single | WT pooled | WT single | WT pooled |
| Number of values | 229 | 176 | 189 | 192 | 178 | 224 |
|  |  |  |  |  |  |  |
| Minimum | 25.2 | 25 | 25 | 25 | 25.71 | 26.32 |
| 25% Percentile | 36.94 | 35.95 | 44.1 | 36.16 | 42.86 | 38.19 |
| Median | 56.59 | 53.21 | 66.67 | 54.17 | 62.2 | 58.06 |
| 75% Percentile | 83.64 | 80.73 | 143.8 | 100 | 100 | 107 |
| Maximum | 54190 | 8100 | 800 | 800 | 500 | 800 |
|  |  |  |  |  |  |  |
| Mean | 456.2 | 134.6 | 127.2 | 99.93 | 84.65 | 87.29 |
| Std. Deviation | 3717 | 619.1 | 147.8 | 122.3 | 66.43 | 83.01 |
| Std. Error | 245.6 | 46.66 | 10.75 | 8.829 | 4.979 | 5.546 |
|  |  |  |  |  |  |  |
| Lower 95% CI of mean | -27.84 | 42.5 | 106 | 82.52 | 74.83 | 76.36 |
| Upper 95% CI of mean | 940.2 | 226.7 | 148.4 | 117.3 | 94.48 | 98.22 |
|  |  |  |  |  |  |  |
| Sum | 104466 | 23689 | 24038 | 19187 | 15068 | 19552 |
|  |  |  |  |  |  |  |
