# Supplementary material for: Human‐Induced Pluripotent Stem Cells Generate Light Responsive Retinal Organoids with Variable and Nutrient‐Dependent Efficiency
Source: Stem Cells. 2018 Aug 13;36(10):1535–51. doi: 10.1002/stem.2883 (PMC6392112; doi:10.1002/stem.2883)
Supplement: Supplementary file 10 — Table S4. (A): Table showing significant single interactions on gene expression for design 1. (B): Table showing two way interactions for design 1. [file STEM-36-1535-s001.docx]

| **MAIN EFFECTS TABLE** | | | | | | | | |
| --- | --- | --- | --- | --- | --- | --- | --- | --- |
| **Target** | **Cell Number (6000 – 12000)** | ***P*** | **BMP4 nM (0.75 - 2.25nM)** | ***P*** | **Lipids (0.5-1.5%)** | ***P*** | **KSR (5-15%)** | ***P*** |
| *VSX2* | -0.67 | 0.001 |  |  |  |  |  |  |
| *RPE65* | -1.96 | 0.001 | 1.38 | 0.01 |  |  |  |  |
| *RCVRN* | -1.71 | 0.001 |  |  | -0.49 | 0.001 | 0.64 | 0.001 |
| *PROX1* | -0.61 | 0.001 |  |  |  |  |  |  |
| *MITF* | -0.63 | 0.001 |  |  |  |  |  |  |
| *MATH5* | -1.29 | 0.001 | 0.33 | 0.01 |  |  |  |  |
| *CRX* | -1.2 | 0.001 |  |  | -0.39 | 0.001 | 0.57 | 0.001 |
| *AP2-α* | 0.8 | 0.01 |  |  |  |  |  |  |

**(A)**

| **(B)**  **2-WAY INTERACTIONS TABLE** | | | | | | |
| --- | --- | --- | --- | --- | --- | --- |
| **Target** | **BMP4*Cell Number** | ***P*** | **Lipids*Cell Number** | ***P*** | **Lipids*BMP4** | ***P*** |
| *RPE65* | -0.63 | 0.05 | -0.515 | 0.05 |  |  |
| *RCVRN* | -0.4 | 0.001 |  |  | 0.35 | 0.001 |
| *PROX1* | 0.33 | 0.001 |  |  |  |  |
| *MATH5* | -0.26 | 0.05 |  |  |  |  |

**Table S4**. **(**A**) Table showing significant single interactions on gene expression for design 1. (**B**) Table showing 2 way interactions for design 1.**
