# Supplementary material for: Human‐Induced Pluripotent Stem Cells Generate Light Responsive Retinal Organoids with Variable and Nutrient‐Dependent Efficiency
Source: Stem Cells. 2018 Aug 13;36(10):1535–51. doi: 10.1002/stem.2883 (PMC6392112; doi:10.1002/stem.2883)
Supplement: Supplementary file 11 — Table S5. (A): Table showing significant single interactions on gene expression for design 2. (B): Table showing two way interactions for design 2. [file STEM-36-1535-s002.docx]

**(A)**

| **MAIN EFFECTS TABLE** | | | | | | |
| --- | --- | --- | --- | --- | --- | --- |
| **Target** | **Cell Number (3000 – 8000)** | ***P*** | **BMP4 nM (2-5nM)** | ***P*** | **CHIR99021 (1.5-6µM)** | ***P*** |
| *VSX2* | 3.77 | 0.001 |  |  |  |  |
| *RPE65* | 0.64 | 0.01 |  |  |  |  |
| *RECOVERIN* | 0.76 | 0.01 |  |  | -1.32 | 0.001 |
| *PROX1* | 0.43 | 0.001 |  |  | -0.37 | 0.01 |
| *MITF* | 0.82 | 0.001 | -0.41 | 0.01 |  |  |
| *MATH5* | 3.21 | 0.001 | -2.04 | 0.05 | -4.6 | 0.001 |
| *CRX* | 1.18 | 0.001 | -0.48 | 0.05 | -0.58 | 0.05 |

**(B)**

| **2-WAY INTERACTIONS TABLE** | | | | |
| --- | --- | --- | --- | --- |
| **Target** | **CHIR99021*SU5402** | ***P*** | **CHIR99021*Cell Number** | ***P*** |
| *RPE65* | -0.42 | 0.05 |  |  |
| *RECOVERIN* |  |  | -0.95 | 0.001 |
| *MITF1* |  |  | 0.41 | 0.01 |
|  |  |  |  |  |

**Table S5. (A) Table showing significant single interactions on gene expression for design 2. (B) Table showing 2 way interactions for design 2.**
